# The Tumor Suppressor Gene, RASSF1A, Is Essential for Protection against Inflammation -Induced Injury

**DOI:** 10.1371/journal.pone.0075483

**Published:** 2013-10-16

**Authors:** Marilyn Gordon, Mohamed El-Kalla, Yuewen Zhao, Yahya Fiteih, Jennifer Law, Natalia Volodko, Anwar Mohamed, Ayman O. S. El-Kadi, Lei Liu, Jeff Odenbach, Aducio Thiesen, Christina Onyskiw, Haya Abu Ghazaleh, Jikyoung Park, Sean Bong Lee, Victor C. Yu, Carlos Fernandez-Patron, R. Todd Alexander, Eytan Wine, Shairaz Baksh

**Affiliations:** 1 Department of Pediatrics, University of Alberta, Edmonton, Alberta, Canada; 2 Department of Biochemistry, University of Alberta, Edmonton, Alberta, Canada; 3 Faculty of Pharmacy and Pharmaceutical Sciences, University of Alberta, Edmonton, Alberta, Canada; 4 The Centre of Excellence for Gastrointestinal Inflammation and Immunity Research (CEGIIR), Faculty of Medicine and Dentistry, University of Alberta, Edmonton, Alberta, Canada; 5 Department of Laboratory Medicine and Pathology, Faculty of Medicine and Dentistry, University of Alberta, Edmonton, Alberta, Canada; 6 Genetics of Development and Disease Branch, National Institute of Diabetes and Digestive and Kidney Diseases, Bethesda, Maryland, United States of America; 7 Department of Pharmacy, Faculty of Science, National University of Singapore, Singapore; 8 Department of Nephrology, University of Alberta, Edmonton, Alberta, Canada; 9 Department of Oncology, Cross Cancer Institute, Edmonton, Alberta, Canada; Massachusetts General Hospital, United States of America

## Abstract

Ras association domain family protein 1A (RASSF1A) is a tumor suppressor gene silenced in cancer. Here we report that RASSF1A is a novel regulator of intestinal inflammation as *Rassf1a^+/−^*, *Rassf1a^−/−^* and an intestinal epithelial cell specific knockout mouse (*Rassf1a^ IEC-KO^*) rapidly became sick following dextran sulphate sodium (DSS) administration, a chemical inducer of colitis. *Rassf1a* knockout mice displayed clinical symptoms of inflammatory bowel disease including: increased intestinal permeability, enhanced cytokine/chemokine production, elevated nuclear factor of kappa light polypeptide gene enhancer in B-cells (NFκB) activity, elevated colonic cell death and epithelial cell injury. Furthermore, epithelial restitution/repair was inhibited in DSS-treated *Rassf1a^−/−^* mice with reduction of several makers of proliferation including Yes associated protein (YAP)-driven proliferation. Surprisingly, tyrosine phosphorylation of YAP was detected which coincided with increased nuclear p73 association, Bax-driven epithelial cell death and p53 accumulation resulting in enhanced apoptosis and poor survival of DSS-treated *Rassf1a* knockout mice. We can inhibit these events and promote the survival of DSS-treated *Rassf1a* knockout mice with intraperitoneal injection of the c-Abl and c-Abl related protein tyrosine kinase inhibitor, imatinib/gleevec. However, p53 accumulation was not inhibited by imatinib/gleevec in the *Rassf1a^−/−^* background which revealed the importance of p53-dependent cell death during intestinal inflammation. These observations suggest that tyrosine phosphorylation of YAP (to drive p73 association and up-regulation of pro-apoptotic genes such as Bax) and accumulation of p53 are consequences of inflammation-induced injury in DSS-treated *Rassf1a^−/−^* mice. Mechanistically, we can detect robust associations of RASSF1A with membrane proximal Toll-like receptor (TLR) components to suggest that RASSF1A may function to interfere and restrict TLR-driven activation of NFκB. Failure to restrict NFκB resulted in the inflammation-induced DNA damage driven tyrosine phosphorylation of YAP, subsequent p53 accumulation and loss of intestinal epithelial homeostasis.

## Introduction

The Ras association domain family protein 1A (RASSF1A) is frequently lost in human cancers by promoter-specific methylation 1. RASSF1A (but not RASSF5A/Nore1A) was previously shown to associate with tumor necrosis factor α receptor 1 (TNF-R1) to promote cell death 2. Although RASSF5A lacks association with TNF-R1, *Rassf5a^−/−^* mice do have defective TNFα responses suggesting that RASSF5A may influence TNFα signaling and apoptosis through alternate pathways such as interactions with sterile-20 like pro-apoptotic kinases, MST1/2 (mammalian Hippo) 3. *Rassf1a^−/−^* mice are viable and fertile, have normal cell cycle parameters and sensitivity to DNA-damaging agents and show no signs of gross genomic instability 4,5. They do, however, develop tumors in response to chemical carcinogens and develop spontaneous tumors by 18 months of age. Several animals had B-cell lymphomas and others had tumors localized within the gastrointestinal tract suggesting an important role for RASSF1A in gastrointestinal physiology 4,5. Recently, it was demonstrated that the *p53^−/−^*/*Rassf1a^−/−^* double knockout mice are viable, fertile and developmentally normal 6. However, the loss of p53 (even in the heterozyogous state) led to >60% of the animals developing tumors in the absence of *Rassf1a* 6. These tumors include several adenocarcinomas and sarcomas localized within the lungs. However, there was no evidence of colonic hyperplasia 6. The *p53^−/−^*/*Rassf1a^−/−^* double knockout mice also revealed enhanced cytokinesis failure and chromosomal abnormalities leading to aneuploidy which suggests importance in mitotic regulation and tumor suppressor function.

It has been determined that 80% of sporadic colorectal cancer (CRC) tumors have somatic and germline mutations within the tumor suppressor gene, adenomatous polyposis coli (APC) 7. In the absence of APC, β-catenin freely translocates to the nucleus where it signals an intracellular cascade resulting in the transcription of hundreds of genes 8–10. The loss of RASSF1A in the context of the *APC^min/+^* mice (*Rassf1a^−/−^APC^min/+^* mice) has been reported to result in increased β-catenin nuclear localization, intestinal adenomas and poorer survival than single knockout littermate controls 11. These data would suggest the importance of the early loss of RASSF1A during premalignant formation of adenocarcinomas that may lead to increased malignant transformation and tumorigenesis. The loss of both APC and RASSF1A function may be key initiating events in the formation of CRC and these observations highlight the importance of RASSF1A in intestinal physiology 10,12.

It has been demonstrated that TNF-R1 is a unique death receptor that can activate a cell death pathway through RASSF1A, triggering Bax activation and mitochondrial-driven apoptosis 1,2,13,14. In addition, TNF-R1 can also promote a pro-inflammatory response through nuclear factor kappa B (NFκB) pathways 15. NFκB is an important mediator of inflammation and the pathogenesis of intestinal inflammation and inflammatory bowel disease (IBD) 16. TLRs can also activate a potent NFκB response and defend against invading pathogens 17. Of the RASSF family members, RASSF6 and 8 were demonstrated to modulate NFκB activity but a mechanism was not proposed 18,19. Recently, Song et al. (2012) demonstrated that RASSF2 can associate with the IKK complex to inhibit NFκB 20 and several reports indicate that RASSF1C and 8 may influence the function of the Wnt/β-catenin pathways, pathways important for the appearance of intestinal tumorigenesis 19,21.

It is currently unknown if RASSF1A can influence NFκB activation via TNF-R1 or TLR pathways. However, epigenetic analysis does reveal promoter specific methylation of RASSF1A resulting in loss of expression in ulcerative colitis (UC) patients 22, a form of IBD. Interestingly, IL-6 can drive epigenetic loss of RASSF1A via DNA methyltransferase 1 (DNMT1) up-regulation 23,24 suggesting that inflammation can drive expression loss of RASSF1A. In this study, we demonstrate an *in vivo* function for RASSF1A in restricting NFκB-dependent inflammation linked to the TLR pathway. Furthermore, we can demonstrate that NFκB-dependent inflammation can modulate epithelial proliferation and the appearance of tyrosine phosphorylation of the Hippo pathway target, Yes-associated protein (YAP). We believe that tyrosine phosphorylated YAP can up-regulate pro-apoptotic genes to drive intestinal epithelial apoptosis. Restricting NFκB-dependent inflammation and tyrosine phosphorylation of YAP will maintain epithelial homeostasis, promote epithelial restitution and allow recovery from inflammation induced injury.

## Experimental Procedures

All animal experiments/husbandry have been approved and follow the guidelines of the Canadian Animal Care and Use Committee and the animal ethics board at the University of Alberta (permit numbers #461 and 639). All animals (except for the *Rassf1a^ IEC-KO^* and *Rassf1a ^IEC-WT^*) were on the C57BL/6 background. Rassf1a ^IEC-KO^ and *Rassf1a ^IEC-WT^* were on the C57BL/6-129 background. Although the loss of *Rassf1a* can lead to tumor formation, the loss of *Rassf5a* (*Rassf5a^−/−^* mice) has not been documented to display an overt phenotype nor tumor formation as they age. We therefore used the *Rassf5a^−/−^* mice as a related control 3. Villin-Cre transgenic mice [B6.SJL-Tg(Vil cre)997Gum/J] was obtained from Jackson Laboratories.

### Genotyping Of Intestinal Specific Knockout Mice

PCR was utilized to detect the conditional allele of Rassf1a (RSF-C/RSF-3), for the deletion of exon 1α of *RASSF1A* (RSF-5/RSF-3) 5 and the presence of the Villin Cre-transgene (IMR1878/IMR1879/IMR0015 and IMR0016) (Jackson Laboratories) as described previously. RSF-3 (reverse primer), 5′-CCA GGC TTC CTT CTC ACT CCT CTG CCG C-3′; RSF-C (forward primer), 5′-CTC GCC CCT GTC AAA GAA AGC TGC TCT GGG GTT CT-3′; RSF-5 (forward primer), 5′-CTC GCC CCT GTC AGA CCT CAA TTT CCC-3′, IMR1878 (Cre Transgene), 5′-GTG TGG GAC AGA GAA CAA ACC-3′; IMR1879 (Cre Transgene), 5′-ACA TCT TCA GGT TCT GCG GG-3′; IMR0015 (internal positive control forward), 5′-CAA ATG TTG CTT GTC TGG TG-3′; IMR0016 (internal positive control reverse), 5′-GTC AGT CGA GTG CAC AGT TT-3′. PCR cycle conditional allele or deleted gene: 94°C for 3 min (1 cycle) followed by 94°C for 30 s, 65°C for 1 min, 72°C for 30 s (30 cycles) with a final cycle of 72°C for 10 min and keep at 4°C till the end. PCR for Cre Transgene, 1 cycle at 94°C for 3 min followed by 35 cycles of 94°C for 30 s, 62°C for 1 min, 72°C for 1.5 min, with a final step of 72°C for 1 min and keep at 4°C till the end. RSF-C/RSF-3 produces a 400 bp fragment for wild-type allele of *Rassf1a* and a 480 bp fragment for conditioned allele. RSF-5/RSF-3 produces a 400 bp fragment only in the mouse tissue that has Exon 1α of *Rassf1a* deleted. The Cre Transgene genotyping produces a 200 bp fragment for the wild-type allele and an 1100 bp fragment for the Cre Transgene.

### Mouse Histology

The descending colon was isolated, fixed in z-Fix (Anatech Ltd) and paraffin-embedded. All inflammation scores were obtained utilizing blinded scoring by a gastrointestinal pathologist (Dr. Aducio Thiesen) based on infiltration of enterocytes, neutrophils, lamina propria cellularity, crypt structure and epithelial hyperplasia (scored as 0 – 2 where 2 =  maximal injury).^25^ Crypt depth was measured using a Leica SP5 confocal microscope at 40X magnification. Immunohistochemistry (IHC) and hematoxylin and eosin (H&E) staining were carried out using standard techniques.

### Isolation Of Mouse Cells

*Splenoctyes* were obtained by excising the spleen and crushing it between two frosted glass slides to generate a single cell suspension. Red cell lysis (8.3 g/L NH_4_Cl, 0.01 M Tris-HCL pH 7.5) was utilized to remove the erythrocytes and the remaining spleen cells (mainly B, T cells and monocytes) were cultured *ex vivo* in RPMI/10% Bovine growth serum/1% Penicillin-Streptomycin. *Bone marrow derived macrophages (BMDM)* were isolated from the femur and grown in DMEM/10% Bovine growth serum/1% Penicillin-Streptomycin for 2–3 days on tissue culture dishes 26. Non-adherent cells (mainly macrophages) were transferred to Petri dishes and grown in DMEM/10% Bovine growth serum/1% Penicillin-Streptomycin containing 20% L cell conditioned media (supernatant from L cells). Macrophages are the only cells that will adhere to the Petri dish. Purity was determined by flow-cytometry analysis using F4/80 and CD4+ markers. *Intestinal crypt cells* were isolated as outlined previously 27. Briefly, colons were flushed with cold 1× PBS, cut open longitudinally and then soaked in 1X PBS with gentle shaking for 20 minutes. The colons were cut into small pieces and incubated with 0.04% sodium hypochlorite for 30 min with gently shaking in a 10 cm^2^ Petri dish. Following incubation, the colon pieces were removed and allowed to shake continuously at room temperature for 30 minutes in a solution containing 1X PBS/1 mM EGTA/1 mM EDTA. Cells were then dislodged by pipetting the tissue up and down using a 25 mL serological pipette until the solution became cloudy. The supernatant was removed (containing the crypt cells) and centrifuged at 3000 rpm for 10 minute to collect the crypt cells. The cells were washed in 1X PBS, transferred to an eppendorf tube, pulse spun to pellet the cells followed by nuclear extraction as previously described 26. Generally, one crypt cell preparation for nuclear extraction was obtained from two colons. Colon protein lysates were prepared by homogenizing in TPER lysis buffer (Fisher Scientific) following isolation.

### Mouse Colonoscopy

Colonoscopy was carried out using a human ureteroscope from Olympus URF [Uretero-Reno Fiberscope] Type P5 within the Center for Excellence in Gastrointestinal Inflammation and Immunity Research (CEGIIR) group at the University of Alberta under isoflurane anesthesia.

### Innate Immunity Analysis

Animals were administered 3% w/v DSS (#160110, molecular weight of 10000, MP Biomedicals) in the drinking water for 7 days followed by recovery for 7 days. They were monitored for: piloerection, bloatednesss, tremors, lack of movement, rectal bleeding and weight loss (all on a scale of 0–5 with 5 being very severe, adapted from Madsen et al.) 25. Animals were euthanized once rectal bleeding became grossly apparent. For weight loss, a score of 0 for no weight loss, 1 if <5% loss, 2 for 5–10% loss, 3 for 10 – 15% loss, 4 for 15 – 20% loss and a score of 5>20% loss in initial body weight. Disease activity indices (DAI) were the sum of all individual scores. All animals were 10 – 12 weeks of age or >25 g in body weight at the beginning of the experiment.

### Gut Permeability Experiments

Following innate immunity activation, animals were starved overnight, FITC-Dextran (0.6 mg/g body weight) was orally administered and 4 hours later blood was collected by cardiac puncture. The resulting serum obtained was analyzed for fluorescein isothiocynate (FITC)-Dextran by fluorimetry (excitation 492 nm; emission, 525 nm; PerkinElmer flurometer). In addition, liver, spleen and mesenteric lymph nodes were removed, homogenized in 1X PBS and 10 µL of the lysate were cultured on MacConkey agar plates to detect Gram-negative bacteria translocated from the gut. Similarly, 10 µL of blood was cultured on MacConkey agar plates.

### *In Vitro* Kinase Assays

MST1: MST1 was immunoprecipitated overnight from 600 µg of TPER homogenized colon lysate (rabbit anti-MST antibody, Cell Signaling#3682) was used as described previously 28. Protein A sepharose beads were used to immobilize the MST1 kinase complex for 1 hour, washed twice with wash buffer and twice with kinase buffer (40 mM HEPES, pH 7.5/20 mM MgCl_2_/20 mM β-glycerophosphate/0.1 mM sodium vanadate). The kinase reaction contained 25 µM ATP/5 µCi 32-P-γ-ATP and 2 µg of histone H2B in 20 µL of kinase buffer. It was carried out for 20 minutes at 30°C, samples were separated by SDS-PAGE, dried down using a gel dryer and autoradiographed. Coomassie blue panel reveals the amount of Histone H2B used in the assay.

c-ABL: ABL was immunoprecipitated overnight from 600 µg of TPER homogenized colon lysate using 1 µg each of anti-c-ABL antibody from BD Bioscience (#554148) and Santa Cruz (sc-121). Protein A sepharose beads were used to immobilize the c-ABL kinase complex for 1 hour, washed twice with wash buffer (50 mM Tris, pH 7.5, 150 mM NaCl, 1% Triton X-100, 1 mM sodium vanadate, 1 mM EDTA) and twice with kinase buffer (30 mM HEPES, pH 7.1/10 mM MgCl_2_/2 mM MnCl_2_/5 mM DTT/10% glycerol/0.2 mM sodium vanadate). The kinase reaction contained 10% glycerol/1 µM ATP/5 µCi 32-P-γ-ATP and 6 µg of lysate from HCT116 cells transiently transfected with FLAG-YAP in 30 µL of kinase buffer. The reaction was carried out for 30 minutes at 30°C, samples were separated by SDS-PAGE, dried down using a gel dryer and autoradiographed. Coomassie blue staining confirmed expression levels of FLAG-YAP (Fig. S7D).

### Nfκb Electromobility Shift Assay (emsa)

This was carried out as previously described 26. Briefly, duplex DNA specific for the NFκB binding site was end-labeled with [γ-^32^P] ATP by using T4 polynucleotide kinase, purified using a G-50 sephadex column (Roche) and allowed to associate with 4 µg of the nuclear extracts containing NFκB for 30 min at room temperature. The oligonucleotide was AAATGTGGGATTTTCCCATGA for crypt cell NFκB analysis (the NFκB binding site from the IL-6 promoter) and TCAGAGGGGACTTTCCGAGAGG for BMDM NFκB analysis (the NFκB binding site from the IL-8 promoter). DNA/protein complexes were then separated by non-denaturing gel electrophoresis, dried onto Whatman filter paper and autoradiographed. Nuclear extracts were prepared using NE-PER (ThermoFisher Scientific) as per manufacturer's instructions). Binding buffer was 100 mM Tris-HCL pH 6.5, 500 mM KCl, 1.2 mM EDTA, 12 mM DTT, 20% glycerol and 1 µg Salmon Sperm DNA. The purity of fractions was confirmed with anti-β-tubulin (cytosolic fraction) or anti-lamin B (nuclear fraction) (data not shown).

### Determination Of Reactive Oxygen Species (ros)

To determine the degree of ROS generated, a fluorometric assay, utilizing the unique intracellular oxidation of 2′,7′-dichlorofluorescin diacetate (DCF-DA), was used. Freshly isolated crypt cells were seeded into 96-well plates in triplicate. Cells were immediately treated with 5 µM DCF-DA and fluorescence was monitored over 45 minutes in the dark. Fluorescence was measured using a Synergy H4 Microplate Reader (Biotek Instruments) set to 37°C. Measurements were made using a 485/20 nm excitation and a 528/20 nm emission filter pair and a gain sensitivity setting of 55%. Readings were made from the bottom every 30 seconds for a total of 45 minutes.

### Statistics

Statistical analyses were performed using one-way ANOVA and Students t-test (two-tailed), as indicated using the GraphPad Prism 5 software. All experiments were carried out at least three times. Error bars in all graphs represent the standard error. Statistics for individual experiments are indicated with the graph or figure legends.

## Results

### Decreased Survival Of *Rassf1a^−/−^* Mice Following Exposure To Dss

**Figure 1:**
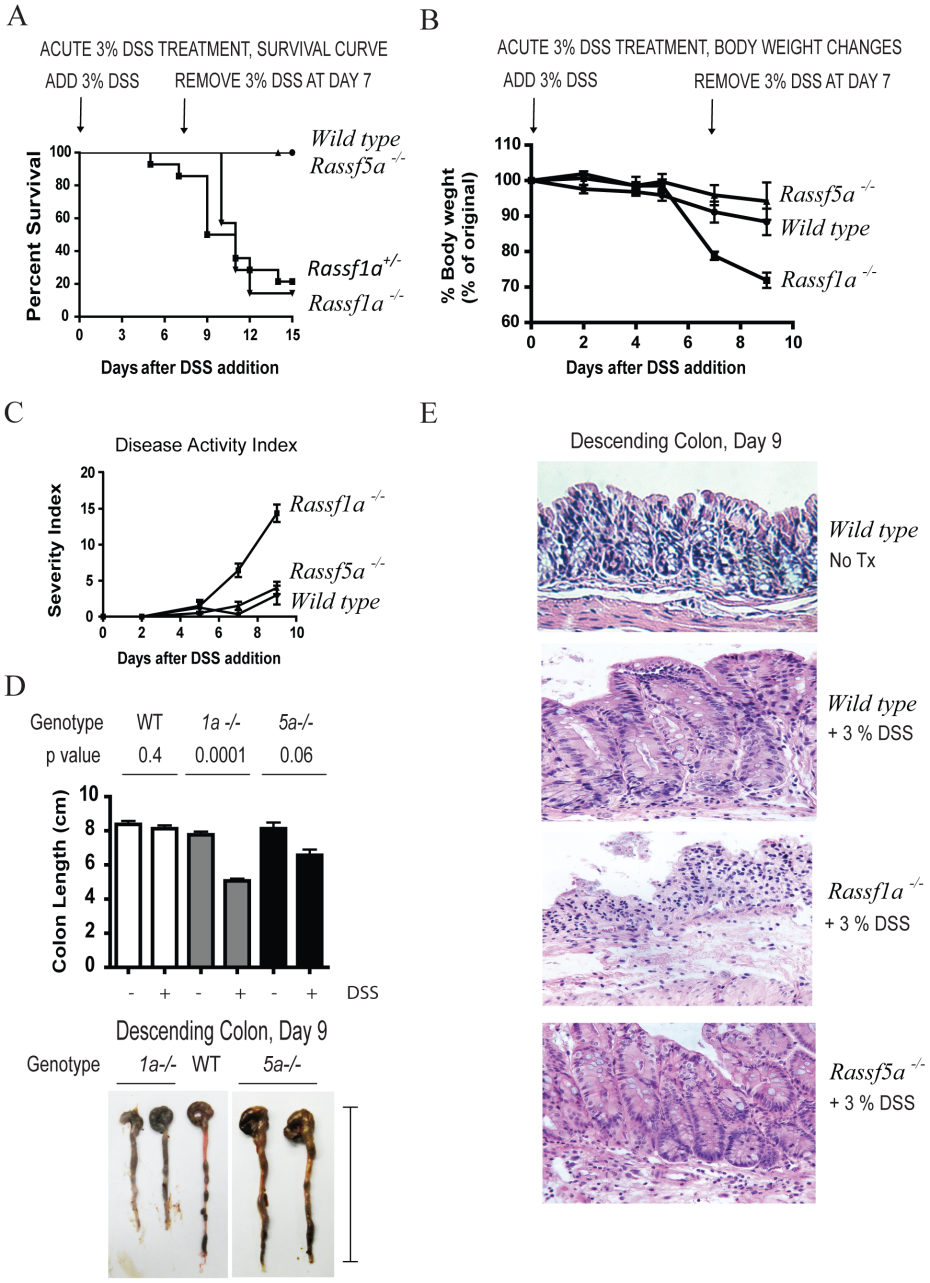
*Rassf1a^−/−^* animals are sensitive to dextran sodium sulphate (DSS) treatment. Mice were subjected to 3% DSS solution followed by day 7 replacement with regular water to allow for recovery. (A) A Kaplan-Meier curve monitoring % survival following DSS treatment. P value <0.0001 (WT/*Rassf5a^−/−^* vs *Rassf1a^−/−^*) and 0.0023 (WT/*Rassf5a^−/−^* vs *Rassf1a^+/−^*); *n* = 12–20. (B) Body weight changes following DSS treatment. P value  = 0.003 (WT/ *Rassf5a^−/−^* vs *Rassf1a^−/−^*); n = 12 – 20. *Rassf1a^+/−^* animals revealed between 20–25% body weight loss (data not shown). (C) Disease activity index (DAI) following DSS treatment. P value  = 0.007 (WT/ *Rassf5a^−/−^* vs *Rassf1a^−/−^*), n = 12–20. *Rassf1a^+/−^* animals revealed DAI between 9–12 (data not shown). (D) Colon length at day 9 of DSS treatment. The indicated p-values represent the difference between −/+DSS treatments within the genotypes; p-value [WT vs *Rassf1a^−/−^* (+DSS)]  = 0.0001; WT vs *Rassf5a^−/−^* (+ DSS)  = 0.01; and *Rassf1a^−/−^* vs *Rassf5a^−/−^* (+DSS)  = 0.0003. A representative picture is shown in the bottom panel indicating how colon length was measured. DSS-treated *Rassf1a^+/−^* revealed a similar loss of colon length (data not shown). (E) A longitudinal cross-section of the descending colon stained with H&E is shown for untreated and DSS-treated animals. Additional images for DSS-treated *Rassf1a^−/−^* are in Fig. S1C as well as for untreated *Rassf1a^−/−^* and *Rassf5a^−/−^* colon sections. All untreated colon sections samples were very similar to untreated colon sections from wild type mice.

Maintaining intestinal homeostasis relies on the ability to control insults from resident microbes in the intestinal lumen by maintaining and preserving intestinal permeability function. DSS is a chemical irritant of the colonic mucosa resulting in epithelial barrier damage, microbial invasion and activation of TLR-expressing epithelial cells triggering NFκB activation. Administration of DSS in the drinking water results in colitis in rodents, mimicking human symptoms of UC. These symptoms include weight loss, diarrhea and rectal bleeding. Upon DSS treatment, *Rassf1a^−/−^* mice became sick within 6 – 9 days post-treatment (Fig. 1A) with reduced activity, piloerection, a general moribund state/hunched posture, lack of grooming, severe rectal bleeding and significantly reduced survival. Wild-type and *Rassf5a^−/−^* mice displayed only mild symptoms of DSS-induced intestinal injury and demonstrated full recovery from DSS exposure (Fig. 1A). *Rassf1a^−/−^* mice revealed >25% loss of body weight (Fig. 1B), increased DAI (Fig. 1C), significantly shortened colon (Fig. 1D) and decreased crypt depth (Fig. S1A) in contrast to wild-type and *Rassf5a^−/−^* mice. Histological sections stained with H&E revealed severe colonic disruption with mucosal/crypt damage (cryptitis and crypt abscesses) in *Rassf1a^−/−^* mice with evident cellular infiltrates composed of immune cells (Fig. 1E and Fig. S1B). DSS-treated wild type and *Rassf5a^−/−^* mice revealed minimal colonic morphology changes and significantly less cellular infiltrates (Fig. 1C). Surprisingly, the *Rassf1a^+/−^* mice also demonstrated similar symptoms to the *Rassf1a^−/−^* mice suggesting haploinsufficiency at the *Rassf1a* locus (Fig. 1A). Since DSS-treated *Rassf1a^+/−^* mice phenocopy the *Rassf1a^−/−^* mice data henceforth is presented for the *Rassf1a^−/−^* mice for most of this article. Genotyping and western blot confirmation of loss of *Rassf1a* is shown in Fig. S1C.

### Dss-Treated *Rassf1a^−/−^* Mice Have Enhanced Inflammation

**Figure 2:**
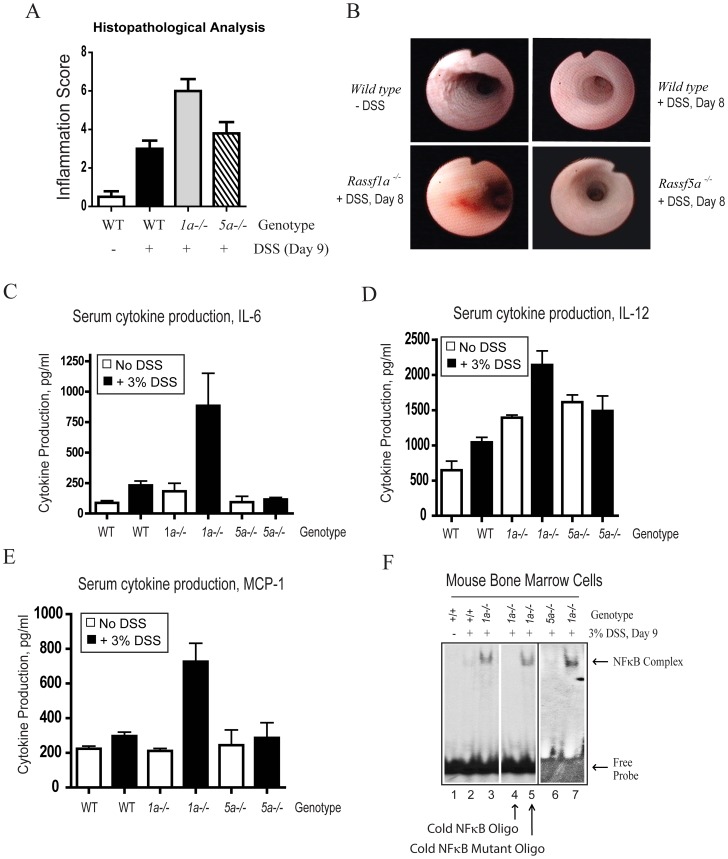
*Rassf1a^−/−^* animals have elevated inflammatory biomarkers following DSS treatment. (A) Inflammation scoring of colon sections stained with H&E. p value of wild type (+DSS) vs *1a^−/−^* is 0.0008 and wild type (+DSS) vs *5a^−/−^* is 0.290 (n = 5 – 10). (B) Colonoscopy images of 3% DSS-treated animals. The *Rassf1a^−/−^* mice (+DSS) were euthanized on day 9 due to disease, whereas all others recovered from 3% DSS treatment. Untreated *Rassf1a^−/−^* or *Rassf5a^−/−^* mice had images similar to wild type untreated mice. (C – E) Blood serum levels of the indicated cytokines. The p value of DSS treated wild type vs *Rassf1a^−/−^* = 0.0001 for IL-6 and MCP-1; 0.005 for IL-12; *n* = 10–14 per group. DSS-treated *Rassf1a^+/−^* revealed as similar increases in IL-6 (Figure 8C) and IL-12 (data not shown). (F) NFκB DNA binding in nuclear extracts derived from bone marrow cells upon DSS treatment. Cold wild type or mutant NFκB oligonucleotide was added to determine the specificity of the observed band (lanes 4 and 5, respectively). Lanes 3 and 7 are the same treatment and genotype but BMDM was obtained from different animals. For (A) and (F), all untreated results for *Rassf1a^−/−^* and *Rassf5a^−/−^* were similar to wild type (untreated).

Histological sections from DSS-treated *Rassf1a^−/−^* colons also revealed mild epithelial hyperplasia as reflected by increased inflammation scores in *Rassf1a^−/−^* treated mice (Fig. 2A). Using mouse colonoscopy, we observed significant DSS-induced injury by day 8 in *Rassf1a^−/−^* versus wild-type or *Rassf5a^−/−^* mice. Reduction of proper colonic striations and disappearance of a normal vascular pattern together with the presence of erythema was observed in the *Rassf1a^−/−^* mice indicating active inflammation (Fig. 2B). Together, these findings suggest that RASSF1A is important for protection against DSS-induced intestinal inflammation.

To determine the mechanism of DSS-induced injury in *Rassf1a^−/−^* mice, levels of inflammatory biomarkers were assessed. Serum levels of IL-6, IL-12 and MCP-1 (but not IL-1β, TNFα, IFNγ or IL-23) were elevated in *Rassf1a^−/−^* mice compared to wild-type or *Rassf5a^−/−^* mice (Fig. 2C – E). Following DSS treatment, NFκB specific activity in bone marrow cells from *Rassf1a^−/−^* animals was significantly higher than in wild-type or *Rassf5a^−/−^* bone marrow cells (Fig. 2F). The detected activity was specific for NFκB (Fig. 2F, lane 4) and lost in the presence of a cold competitor oligonucleotide or an NFκB mutant DNA binding oligonucleotide (Fig. 2F, lanes 4 and 5, respectively). We further characterized inflammation injury in *Rassf1a^−/−^* versus wild-type mice by measuring myeloperoxidase (MPO, a glycoprotein rapidly released by activated neutrophils) and hyaluronic acid (HA) release. Following DSS-induced intestinal injury, HA synthase (HAS) upregulates HA production in macrophages and lamina propria lymphocytes 29, targeting TLR2 and TLR4, and subsequently promoting TLR4/2-driven NFκB activity 30. We observed a significant increase in both serum and colon tissue levels of MPO (Fig. S1D) and serum HA levels (Fig. S1E) in DSS-treated *Rassf1a^−/−^* mice (a 2 – 3 fold increase in both markers was observed). Elevated HA levels may indicate a sustained need to activate TLR4-driven protective responses to promote epithelial repair in the continued presence of inflammation-induced damage 30. In contrast, elevation of MPO may be detrimental to the recovery from DSS-induced injury and may have significantly contributed to the decreased survival of *Rassf1a^−/−^* mice.

### *Ex Vivo* Analysis Of *Rassf1a^−/−^* Stimulated Bone Marrow Cells Reveal Abnormal Nfκb Regulation

**Figure 3:**
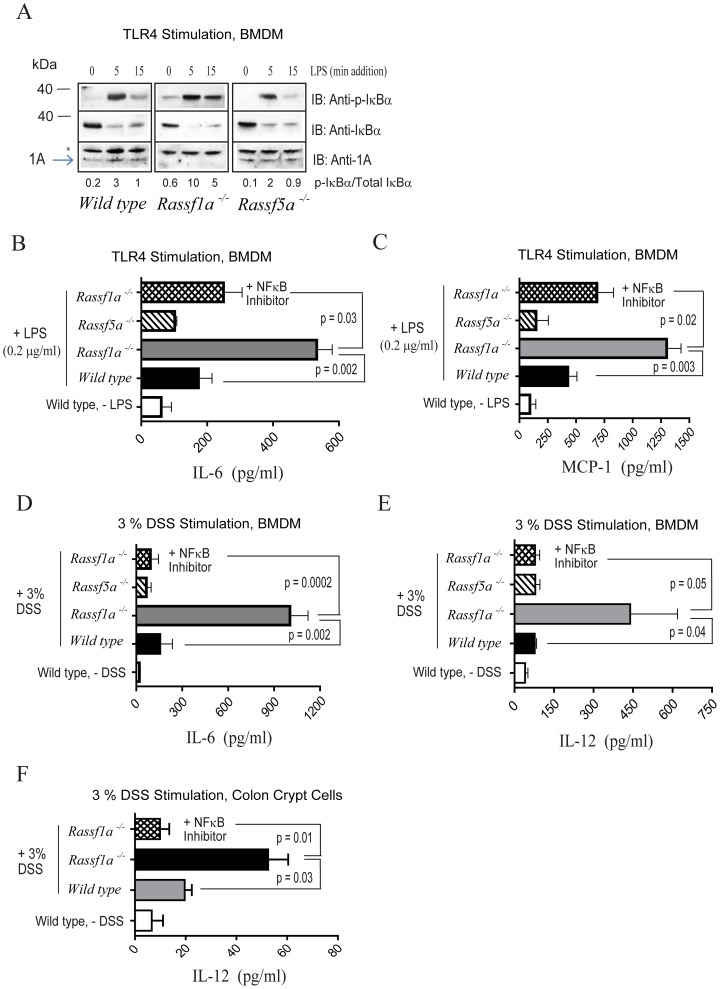
*Rassf1a^−/−^* animals show increased levels of biomarkers of inflammation. (A) *Ex vivo* analysis of bone marrow derived macrophages was carried out for the presence of phospho-(p)-IκBα (A, repeated in triplicate) and serum pro-inflammatory cytokine production (B – E, n = 6–10). LPS was for 5 hours (B – C) and 3% DSS for 16 hours in media (D – E). For (B – E) n = 4. (F) Colon epithelial crypt cells were isolated, cultured and stimulated the same day with 3% DSS for 16 h. IL-6 secreted into the supernatant was quantitated (n = 4). Stimulation of colon epithelial crypt cells from the *Rassf5a^−/−^* mice was not carried out. Stimulation of isolated colon epithelial crypt cells with LPS produced similar results (data not shown). For (B – F) all untreated results for *Rassf1a^−/−^* and *Rassf5a^−/−^* were similar to wild type (untreated). The NFκB inhibitor utilized was 10 µM methoxyresveratrol.

Our *in vivo* results implicate NFκB in intestinal inflammation in DSS-treated *Rassf1a^−/−^* mice. Activation of intestinal epithelial cells (IECs) generally results in signals transmitted to the bone marrow and lymph nodes, resulting in activation of macrophages and lymphocytes (that is, the adaptive immune response). The reported changes in NFκB DNA-binding activity in bone marrow cells (Fig. 2F) suggested that DSS-driven activation of IECs can be transmitted to the bone marrow to promote an adaptive immune response and amplify NFκB activity. Alternatively, the intrinsic loss of RASSF1A in hematopoietic cells could also cause hyperactivation of NFκB in the immune cells and reflect an important role for RASSF1A in these cells. This issue was addressed by culturing bone marrow derived macrophages (BMDM), splenocytes or crypt cells *ex vivo* (from untreated animals) and stimulating them with lipopolysaccharide (LPS) or DSS (both activators of TLR4) 29,30. Normally, NFκB is inhibited by IκBαwhich is phosphorylated and targeted for degradation following LPS or DSS stimulation, allowing nuclear translocation and activation of NFκB. Analysis in *Rassf1a^−/−^* BMDM revealed a sustained increase in IκBα phosphorylation following LPS stimulation (even up to 2 hours post LPS-addition [data not shown]); while both wild-type and *Rassf5a^−/−^* BMDM showed transient expression of IκBα phosphorylation (Fig. 3A). This indicated that there was an inherent failure of BMDM from *Rassf1a^−/−^* mice to downmodulate NFκB activity when compared to LPS or DSS-treated BMDM from wild type or *Rassf5a^−/−^* mice.

Not surprisingly, *ex vivo* LPS-stimulated *Rassf1a^−/−^* BMDM revealed elevated production of NFκB-regulated pro-inflammatory elements (IL-6 and MCP-1) (Fig. 3B – C) as well as IL-12 and TNFα (Fig. S2A – B). Both IL-6 and IL-12 were also secreted from *Rassf1a^−/−^* BMDM upon treatment with 3% DSS (Fig. 3D – E). IL-10 (an anti-inflammatory cytokine) and MCP-1 were unchanged upon DSS stimulation (Fig. S2C and data not shown, respectively) nor was IL-10 changed with LPS stimulation (data not shown). The elevated production of IL-6 and IL-12 (upon LPS or DSS stimulation) and MCP-1 (upon LPS stimulation) can be inhibited by utilizing the NFκB inhibitor, (E)-2-Fluoro-4′-methoxystilbene (methoxyresveratrol, IC_50_ = 150 nM) (Fig. 3B – E and Fig. S2A). Methoxyresveratrol has been demonstrated to effectively inhibit LPS-induced COX-2 mRNA production as well as TNFα-driven NFκB activation in cultured cells 31. These data strongly suggested that NFκB activity is driving cytokine production *ex vivo* in BMDM cells from *Rassf1a^−/−^* mice upon LPS or DSS stimulation. In addition, it also suggests that RASSF1A may function to restrict NFκB activation within the TLR4 pathway. We also observe the abnormal activation of NFκB in *ex vivo* stimulated colon crypt cells from *Rassf1a^−/−^* mice upon DSS stimulation that can also be inhibited by methoxyresveratrol (Fig. 3F). No changes were observed between wild-type/*Rassf5a^−/−^* mice versus *Rassf1a^−/−^* mice for the levels of secreted IL-1β, IFNγ? nor IL-23 from *ex vivo* LPS or DSS-stimulated BMDM (data not shown). BMDM stimulation with the TLR9 activator, CpG DNA (but not the TLR2 ligand PAM_3_CSK_4_) resulted in the elevated production of TNFα (Fig. S2D and data not shown respectively) and we can also observe increased cytokine/chemokine production in isolated *Rassf1a^−/−^* splenocytes stimulated with CpG DNA (Fig. S2D) or LPS (Fig. S2E – F). Taken together, our *ex vivo* data suggests that RASSF1A may have a universal role in modulating cytokine/chemokine production in immune cells as well as in epithelial cells and that RASSF1A may influence both TLR4 and TLR9 pathways linked to NFκB activation.

### Rassf1a Regulates Intestinal Epithelial Homeostasis Following Dss-Induced Inflammation

**Figure 4:**
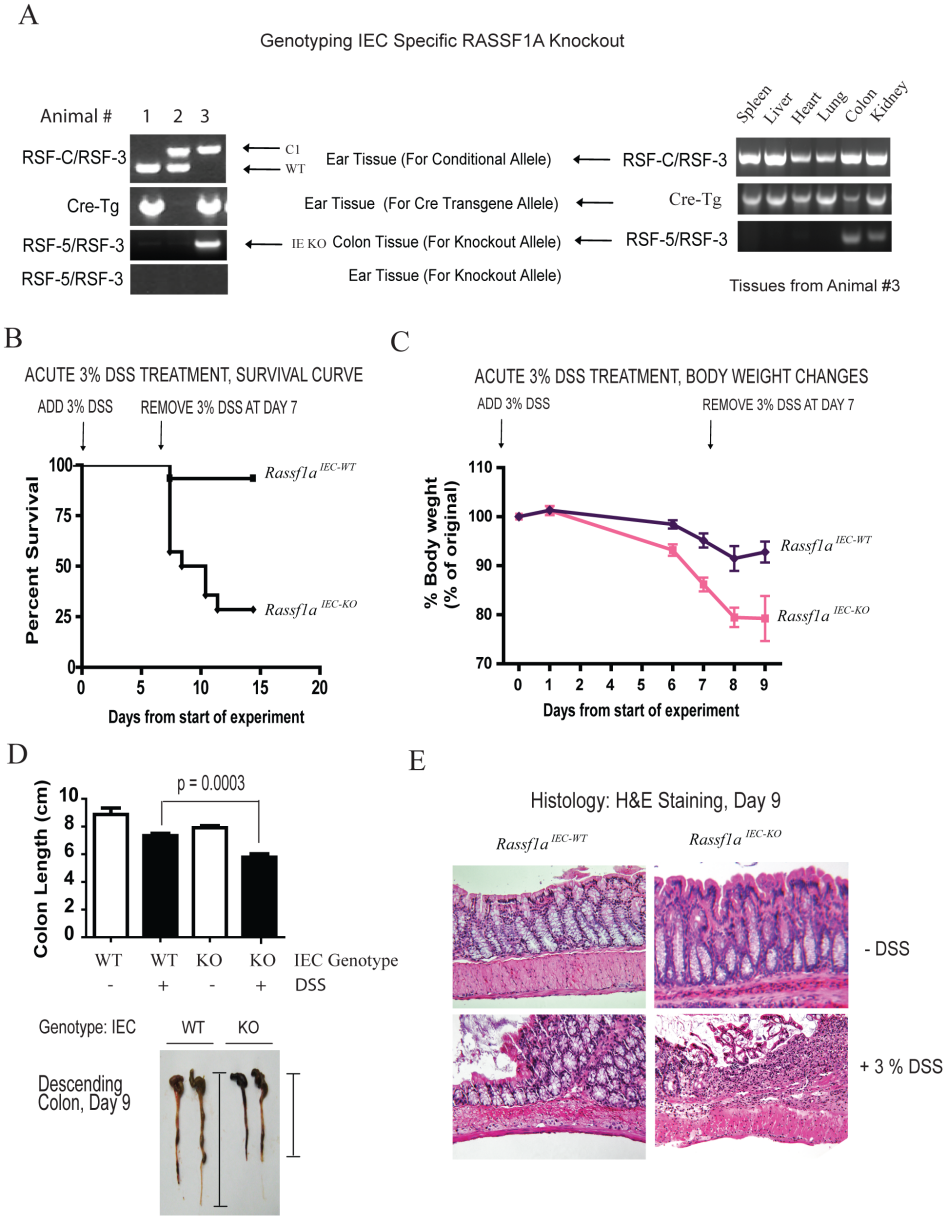
RASSF1A intestinal cell-specific knockout (*Rassf1a^IEC-KO^*) phenocopy *Rassf1a^−/−^* mice following DSS-induced inflammation. (A) PCR detection of the conditional LoxP-1A allele and knockout allele for 1A upon Cre transgene expression in *Rassf1a^IEC-KO^* animals. **Left panel,** As an example, animal #3 contained the IEC-specific knockout, as the conditional allele (c1) and the Cre transgene (Cre-Tg) were observed in the ear tissue and colon (see Methods S1 for further details). C1 =  conditional allele; WT =  wild-type allele. **Right panel,** genotyping in various mouse tissues. (B) Reduced survival of *Rassf1a^IEC-KO^* animals exposed to 3% DSS solution. P value [*Rassf1a^IEC-WT^* vs *Rassf1a^IEC-KO^*] = 0.001, *n* = 12. (C) Decreased body weight change following DSS treatment. P value  = 0.001 (*Rassf1a^IEC-WT^* vs *Rassf1a^IEC-KO^*), *n* = 12. (D) Shortened colon length following DSS-treatment of *Rassf1a^IEC-KO^* vs *Rassf1a^IEC-WT^* mice on day 9. P value (*Rassf1a^ IEC-WT^* vs *Rassf1a^- IEC-KO^*)  = 0.003, n = 11. **Bottom,** Representation of how colon length was measured. (E) Longitudinal cross-section of the descending colon stained with H&E from untreated and DSS-treated *Rassf1a^IEC-WT^* and *Rassf1a^IEC-KO^* mice revealed severely disrupted colonic histology.

**Figure 5:**
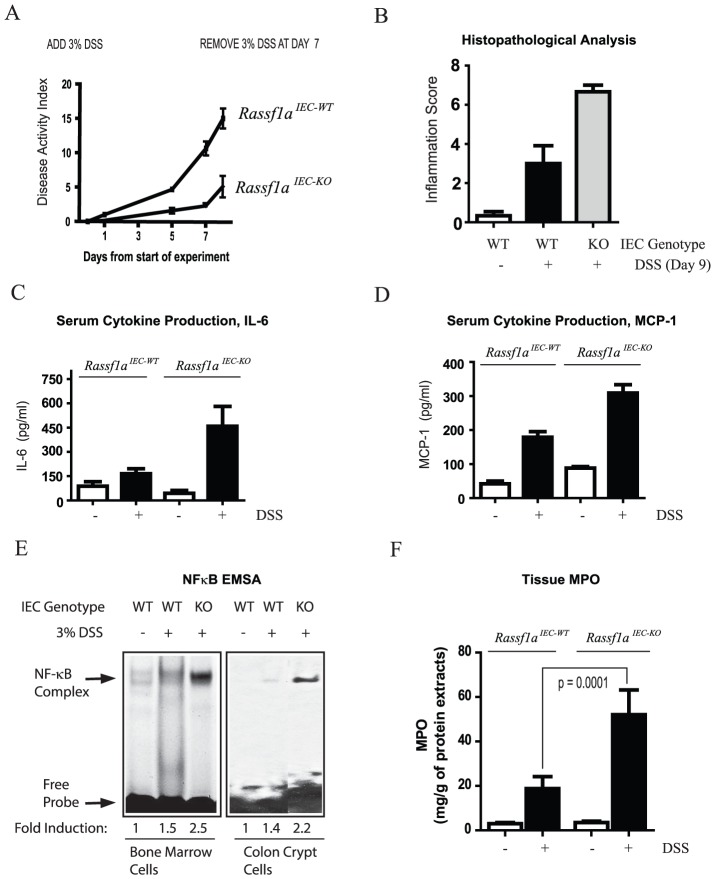
RASSF1A intestinal cell-specific knockout (*Rassf1a^IEC-KO^*) animals have increased levels of inflammatory biomarkers. (A) DAI for survival curve in Fig. 4B. P value  = 0.0001 (*Rassf1 ^IEC-WT^* and *Rassf1a^IEC-KO^*), n = 12. (B) Inflammation score of the indicated genotypes. P value (*Rassf1a^IEC-WT^* and *Rassf1a^IEC-KO^*)  = 0.02. (C and D) Blood serum levels of IL-6 and MCP-1 are shown (day 9). P values (*Rassf1a^IEC-WT^* and *Rassf1a^IEC-KO^*)  = 0.03 for IL-6 and 0.0003 for MCP-1 (*n* = 6–10). (E) NFκB DNA binding activity in nuclear extracts as indicated (day 9). P values for quantitation results  = 0.010 (n = 7) for bone marrow cells and 0.0005 (n = 7) for crypt cells. (F) Tissue MPO activity as determined by ELISA (day 9), *n* = 8 – 10. For (B) and (E) all untreated results for *Rassf1a^IEC-KO^* were similar to *Rassf1a^IEC-WT^* (untreated).

In order to specifically explore the role for RASSF1A in intestinal physiology, an intestinal epithelial cell specific knockout to RASSF1A, *Rassf1a^IEC-KO^*, was generated by mating *Rassf1a-LoxP* conditional mice 5 with the Villin-Cre transgenic mouse and the knockout allele detected by PCR (Fig. 4A). Villin is an intestinal-related cytoskeletal protein that is associated with brush border microvilli. RASSF1A knockout was confirmed by PCR utilizing genomic DNA from colonic sections as previously published 5. Similar to the *Rassf1a^−/−^* mice, DSS-treated *Rassf1a^IEC-KO^* animals displayed <25% survival (Fig. 4B) with significant loss of body weight (Fig. 4C), colon length (Fig. 4D), disrupted colonic crypts and decreased crypt depth (Fig. 4E and Fig. S3A), severe disease activity indices (Fig. 5A) and elevated histopathological scores (Fig. 5B). In addition, DSS-treated *Rassf1a^IEC-KO^* animals had elevated IL-6 and MCP-1 (Fig. 5C – D), increased NFκB DNA binding activity in *Rassf1a^ IEC-KO^* bone marrow cells and colon crypt cells (Fig. 5E) and significant increases in colonic tissue MPO (Fig. 5F) and colonic tissue HA levels (Fig. S3B). Taken together, these data strengthen the protective role of RASSF1A in restricting unwanted NFκB and following DSS-induced inflammation injury.

Not surprisingly, *ex vivo* analysis of LPS or DSS stimulated BMDM or splenocytes from *Rassf1a^IEC-KO^* mice revealed a wild-type like response to LPS or DSS (compare results in Fig. S3C – D versus Fig. 3B – E). This is consistent with RASSF1A only being deleted in IECs and not in immune cells in the *Rassf1a ^IEC-KO^* mice. It is thus the loss of RASSF1A within the intestinal epithelial cells that is promoting intestinal inflammation and amplifying the response by activating BMDM. The elevated responses and high cytokine production possibly explains the poor survival of *Rassf1a* knockout mice. RASSF1A may function to restrict NFκB activation in both colon epithelial and immune cells in order to modulate the inflammatory response. It should be noted that in all *Rassf1a^IEC-KO^* animals, we detected the knockout allele in the kidney as well (Fig. 4, right panel) since villin is also expressed in the epithelia of renal proximal tubules 32. However, RASSF1A immunoblotting revealed the complete loss of RASSF1A from IEC cells but only partial loss of RASSF1A in kidney lysates from *Rassf1a^IEC-KO^* mice (Fig. S3E). Importantly, serum creatinine, systolic blood pressure and renal morphology were similar in *Rassf1a^IEC-KO^* and wild-type mice before or after exposure to DSS (Fig. S4A – C). This excludes renal failure as the cause of the poor survival of *Rassf1a^IEC-KO^* animals upon DSS treatment.

### Dss-Treated *Rassf1a* Knockout Mice Have Increased Gut Permeability

**Figure 6:**
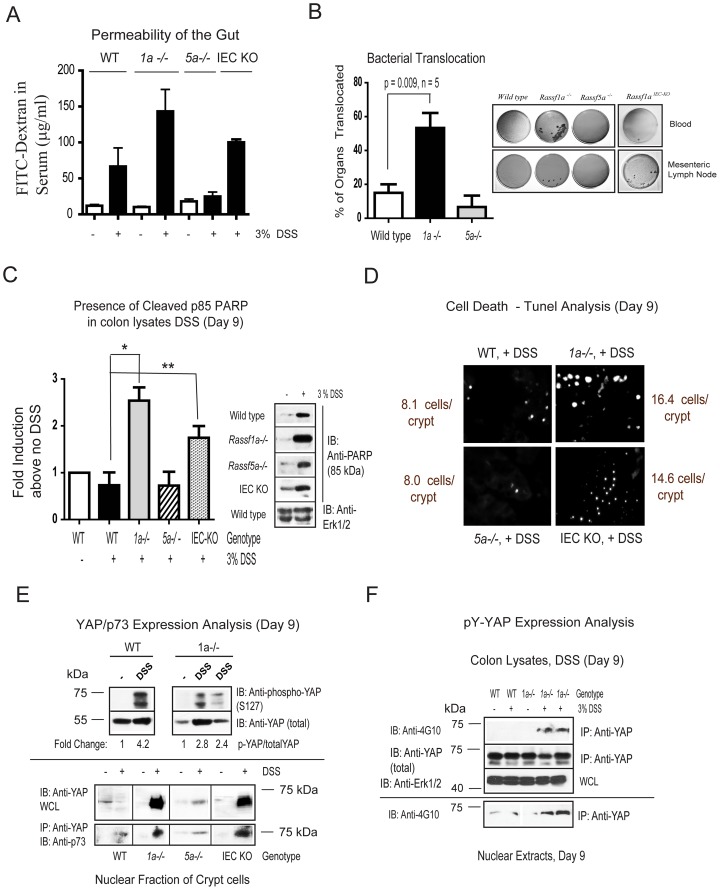
Loss of RASSF1A results in altered intestinal homeostasis. (A) Gut permeability of the indicated animals was determined by FITC-dextran fluorimetry on day 9 post-DSS addition. P values (all + DSS): wild-type vs *Rassf1a^−/−^* = 0.007; wild-type vs *Rassf5a^−/−^* = 0.4; *Rassf1a^−/−^* vs *Rassf5a^−/−^* = 0.005, wild-type vs *Rassf1a^IEC-KO^* = 0.0001, n = 6 – 10 per group. (B) **Right panel**, bacterial translocation to blood and mesenteric lymph nodes as indicated (liver and spleen also revealed some translocation). **Left panel**, histogram plot of the percentage of these four areas colonized with bacteria. (C) Analysis for PARP cleavage was carried out as indicated on day 9 post-DSS treatment. “*” p value  = 0.0028 and “**”  = 0.0295, n = 5 – 7 for all genotypes and treatments. P value wild-type (+DSS) vs *Rassf5a^−/−^* (+DSS)  = 0.989. (D) TUNEL positive staining (bright dots) was carried out as a late marker of cell death. These pictures are a representative of three independent histological sections from each genotype and treatment. The mean number of cells/crypt shown was counted 6 times. P value wild-type (+DSS) vs *Rassf1a^−/−^* or *Rassf1a^IEC-KO^* (+DSS)  = 0.002 and 0.04 respectively. (E) Colon lysates (top panel) or nuclear fraction of isolated crypt cells (bottom panel) from the indicated genotypes were used to detect total or phosphoserine (S)127 YAP, total YAP and p73 in the nuclear fractions. For the top panel, the +DSS lanes are colon lysates from different DSS-treated animals. All treatments and genotypes had similar levels of total p73 following total lysate analysis by immunoblotting (data not shown). (F) Detection of total or pY-YAP as indicated. Purity of nuclear and cytoplasmic fractions are shown in Figure S6E. For (C – D) all untreated results for *Rassf1a^−/−^*, *Rassf5a^−/−^* and *Rassf1a^IEC-KO^* (untreated) were similar to wild type (untreated).

Epithelial barrier breakdown and increased permeability of the gut occurs during severe inflammation and IBD and are associated with increased production of cytokines 33,34. Fortunately, IEC layer integrity is rapidly re-established by epithelial restitution, wound healing and/or increased epithelial proliferation after inflammation-induced injury in order to maintain a healthy gut. These events prevent direct exposure of lamina propria immune cells to the intestinal microflora and a harmful inflammatory response 35. Thus, the epithelial integrity of DSS-treated *Rassf1a^−/−^* mice was examined, assessing cell repair and intestinal permeability. Following a 7 day treatment with DSS and a one day recovery period with regular water, animals were administered FITC-dextran by oral gavage. Serum levels of FITC-dextran were significantly higher in DSS-treated *Rassf1a^−/−^* and *Rassf1a^IEC-KO^* mice versus wild-type/*Rassf5a^−/−^* mice, suggesting a role for RASSF1A in maintaining permeability during the recovery phase of DSS-induced inflammation injury (Fig. 6A). Interestingly, spleen, liver and mesenteric lymph node extracts from DSS-treated *Rassf1a* knockout animals revealed >50% bacterial translocation also indicating increased gut permeability (Fig. 6B). DSS-treated *Rassf1a* knockout mice thus have an impaired ability to contain commensal bacterial in the colon upon inflammation-induced insult.

### Defective Epithelial Restitution In *Rassf1a* Knockout Mice Following Dss-Induced Injury

The increased epithelial permeability and decreased survival of DSS-treated *Rassf1a^−/−^* mice could be explained by the damage caused by disruption of the epithelial barrier (and damaged paracellular space), elevated cell death (apoptosis) or defects in epithelial cell repair and lack of crypt cell proliferation 36. Due to increased colonic permeability and mucosal/crypt damage in *Rassf1a^−/−^* and *Rassf1a^IEC-KO^* mice following DSS insult (Fig. 1D – E and Fig. 4D – E) we speculate that RASSF1A may influence pathways involved in epithelial restitution and/or cell repair to regain normal epithelial architecture. Proliferating cell nuclear antigen (PCNA) staining of tissue colon sections from *Rassf1a^−/−^* and *Rassf1a^IEC-KO^* mice treated with DSS confirmed reduced crypt cell proliferation versus wild-type or *Rassf5a^−/−^* (Fig. S5A). We also observed significant PARP cleavage (a marker of late apoptosis) in colon lysates (Fig. 6C) and increased TUNEL-positive staining in colonic sections (a marker of condensed nuclei and indicative of apoptosis) (Fig. 6D) from DSS-treated *Rassf1a^−/−^* and *Rassf1a^IEC-KO^* mice. The substantial increase in apoptosis in DSS-treated *Rassf1a^−/−^* and *Rassf1a^IEC-KO^* mice is likely a significant contributing factor to the decreased survival observed for these mice in Fig. 1A and 4B.

### Dss-Treated *Rassf1a^−/−^* Mice Triggers Tyrosine Phosphorylation Of Yes-Associated Protein (yap) Following Dss-Induced Injury

RASSF1A can normally promote cell death via TNF-R1 and the loss of RASSF1A should result in decreased cell death. We, in fact, can detect enhanced cell death by PARP and TUNEL analysis (Fig. 6C – D). We thus explored alternative explanations for how the loss of RASSF1A may result in increased intestinal apoptosis. RASSF1A is a component of the Rassf/Salvador/Hippo pathway that can modulate cell death, organ size and cellular proliferation 37–39. It is thought that in mammalian cells, RASSF1A can function to activate MST1/2, resulting in the activation of LATS and downstream serine phosphorylation events 40. Activated LATS can lead to the inactivation (by cytoplasmic re-localization and retention) of the Yes-associated protein (YAP), a key diver of proliferation (known as *Yorkie* [*Yki*] in *Drosophila*) linked to the TEAD family of transcription factors 41 Removal of either *Yki* from intestinal stem cells in *Drosophila* 42 or YAP in *Yap^−/−^* mice 43 revealed poor survival and decreased epithelial cell proliferation in response to DSS treatment (characteristics similar to what we have observed). However, we are arguing for the important role of RASSF1A in restricting NFκB and downstream effects that hyperactivation of NFκB can cause. Although inflammation was not investigated in either of these studies, it does suggest an important role for both RASSF1A and YAP in modulating crypt cell proliferation and survival following DSS-induced inflammation injury.

In the absence of RASSF1A, there was a significant reduction in S127 phosphorylation of YAP (Fig. 6E, top panel) and enhanced nuclear presence of p73 associated YAP (Fig. 6E, bottom panel). Reduced S127-phosphorylated YAP and increased nuclear presence of YAP would be expected to result in increased YAP-driven transcription to drive proliferation of intestinal crypt cells in the canonical Hippo pathway. Enhanced proliferation can be observed in wild-type mice upon DSS treatment but not in DSS-treated *Rassf1a^−/−^* and *Rassf1a^IEC-KO^* mice (Fig. S5A). DSS-treated *Rassf1a^−/−^* and *Rassf1a^IEC-KO^* mice clearly revealed enhanced nuclear levels of YAP but reduced crypt proliferation (as determined by PCNA staining in Fig. S5A). However, increased crypt apoptosis (as determined by elevated PARP cleavage and TUNEL staining) can be observed in line with reduced crypt proliferation (Fig. 6C – D). Furthermore, MST kinase activity was dramatically reduced in DSS-treated of *Rassf1a^−/−^* and *Rassf1a^IEC-KO^* mice (Fig. S5B) in agreement with enhanced nuclear presence of YAP.

**Figure 7:**
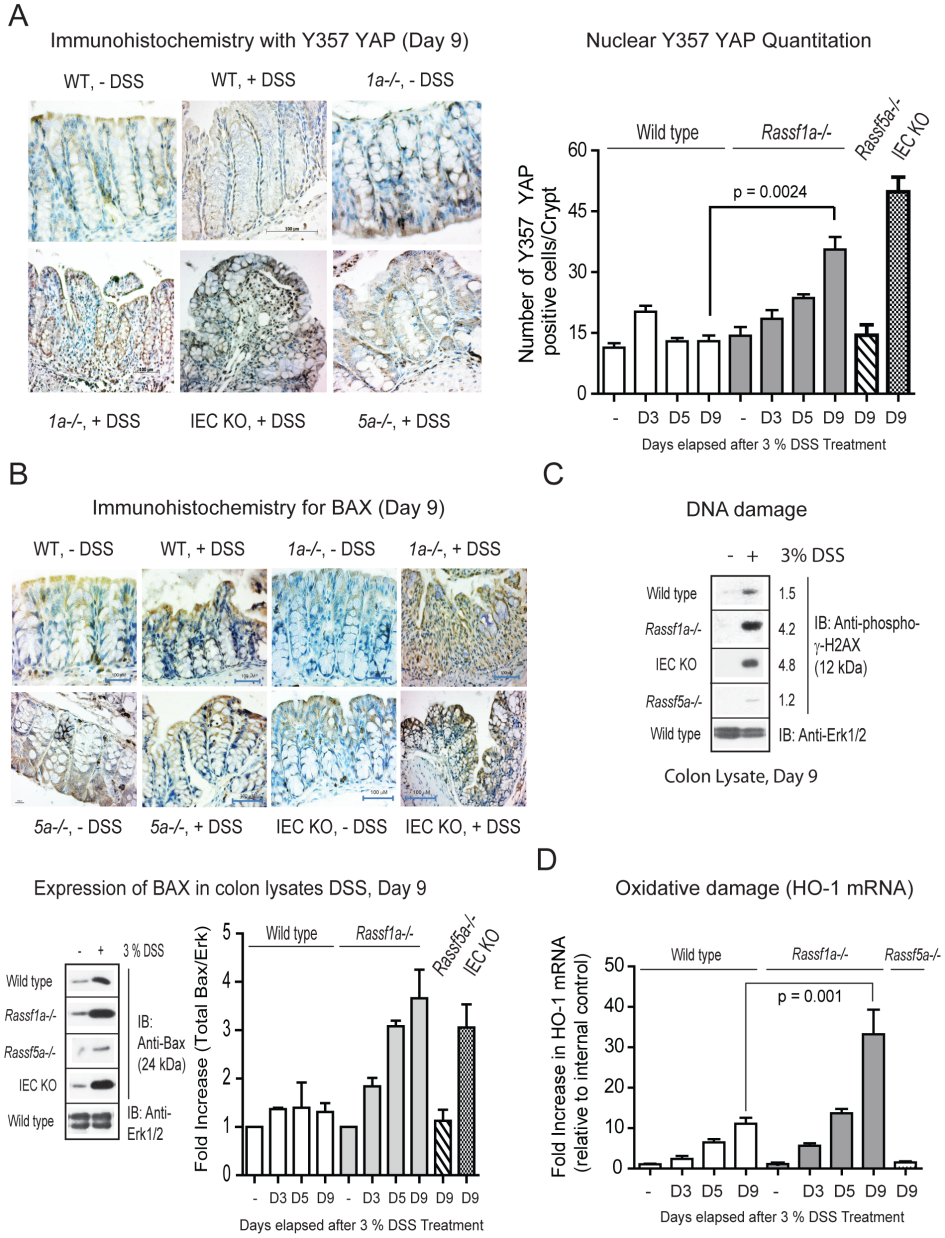
*Rassf1a^−/−^* mice fail to recover following DSS treatment due to abnormal YAP signaling. (A) Phosphotyrosine (pY)-YAP immunohistochemistry (IHC) with anti-Y357 YAP was carried out on colon sections as indicated. **Left panel**, IHC of colon sections using anti-Y357 YAP. **Right panel**, 1000 cells were scored for nuclear presence of YAP Y357. P value wild-type (+DSS) vs *Rassf5a^−/−^* (+ DSS)  = 0.396. (B) **Top panel**, analysis of Bax expression by IHC was carried out as indicated on colonic tissue sections on day 9 post-DSS treatment. **Bottom panel**, expression of Bax in colon lysates from day 9 DSS-treated animals as indicated (fold induction of the immunoblotting results are graphed in the right panel). For graph, “*” p-value  = 0.0093 and “**”  = 0.041, n = 5 – 7 for all genotypes and treatments. P value wild-type (+DSS) vs *Rassf5a^−/−^* (+DSS)  = 0.546. (C) Analysis for the DNA damage marker, phospho-γ-H2AX, following *in vivo* DSS insult in colon lysates (experiment repeated twice with similar results). Numbers represent fold induction relative to phospho-γ-H2AX levels in untreated wild type animals. (D) Real time PCR analysis for an oxidative damage marker hemexogenase-1 (HO-1) utilizing colonic mRNA (n = 4 for all and D designates the day that the tissues were harvested). P value of wild type (+DSS) versus *Rassf5a^−/−^* is 0.027 (Day 3), 0.006 (day 5) and 0.0008 (day 9) with n = 4 – 6 per day per sample. The *Rassf1a^IEC-KO^* result was comparable to the result in the DSS treated *Rassf1a^−/−^* mice (data not shown).

Further detailed analysis revealed increased tyrosine phosphorylation (pY) of YAP in DSS-treated *Rassf1a^−/−^* mice in colon lysates/nuclear fractions (Fig. 6F) and in colonic sections (Fig. 7A). A clear difference in pY-YAP between DSS-treated wild type and *Rassf1a^−/−^* mice was observed as early as day 5 post DSS addition that continued to increase towards day 9 (Fig. 7A, right panel). Previous studies have suggested a functional role for pY-YAP/p73 complex to ultimately drive pro-apoptotic gene expression, especially Bax 44,45. In support of these arguments, we detected enhanced nuclear presence of a pY-YAP/p73 complex (Fig. 6E, bottom panel), increased Bax expression by immunohistochemistry and immunoblotting (Fig. 7B) in intestinal crypt cells from DSS-treated *Rassf1a^−/−^* and *Rassf1a^IEC-KO^* mice but not in samples from wild-type or *Rassf5a^−/−^* treated mice. This suggests that a pY-YAP/p73 driven up-regulation of Bax may promote intestinal cell death leading to increased gut permeability, lack of effective epithelial repair and poor survival of DSS-treated *Rassf1a^−/−^* and *Rassf1a^IEC-KO^* mice.

Previous studies have suggested a functional role for c-Abl-driven tyrosine 357 phosphorylation of YAP in response to DNA damage 46,47. This resulted in the formation of a pY-YAP/p73 complex to drive pro-apoptotic gene expression, especially Bax 44,45. We detected enhanced Y357 phosphorylation in *Rassf1a* knockout mice using a Y357 YAP antibody (Fig. 6F and Fig. 7A). In addition, we also observed increased DNA damage as early as day 3 (Fig. 7C and Fig. S5C), oxidative damage by day 5 – 9 (Fig. 7D) as well as significant ROS production in colon samples from *in vivo* DSS-treated *Rassf1a* knockout mice (Fig. S5D). DNA and oxidative damage of colonic cells have been characterized as significant factors in the pathogenesis of IBD 48,49. Similar to IBD patients, inflammation-induced injury in our *Rassf1a* knockout mice has associated DNA and oxidative damage. Since we can observe DNA damage as early at day 3, we speculate that this may be driving the tyrosine phosphorylation of YAP observed by day 3 – 5 (Fig. 7A). YAP tyrosine phosphorylation following DSS-induced inflammation injury will in turn drive colonic cell death (via p73 association and transcriptional activation of cell death genes) and poor survival of DSS-treated *Rassf1a* knockout animals.

### Imatinib Can Protect *Rassf1a^+/−^* Mice But Not *Rassf1a^−/−^* Mice From Dss-Induced Inflammation Injury

**Figure 8:**
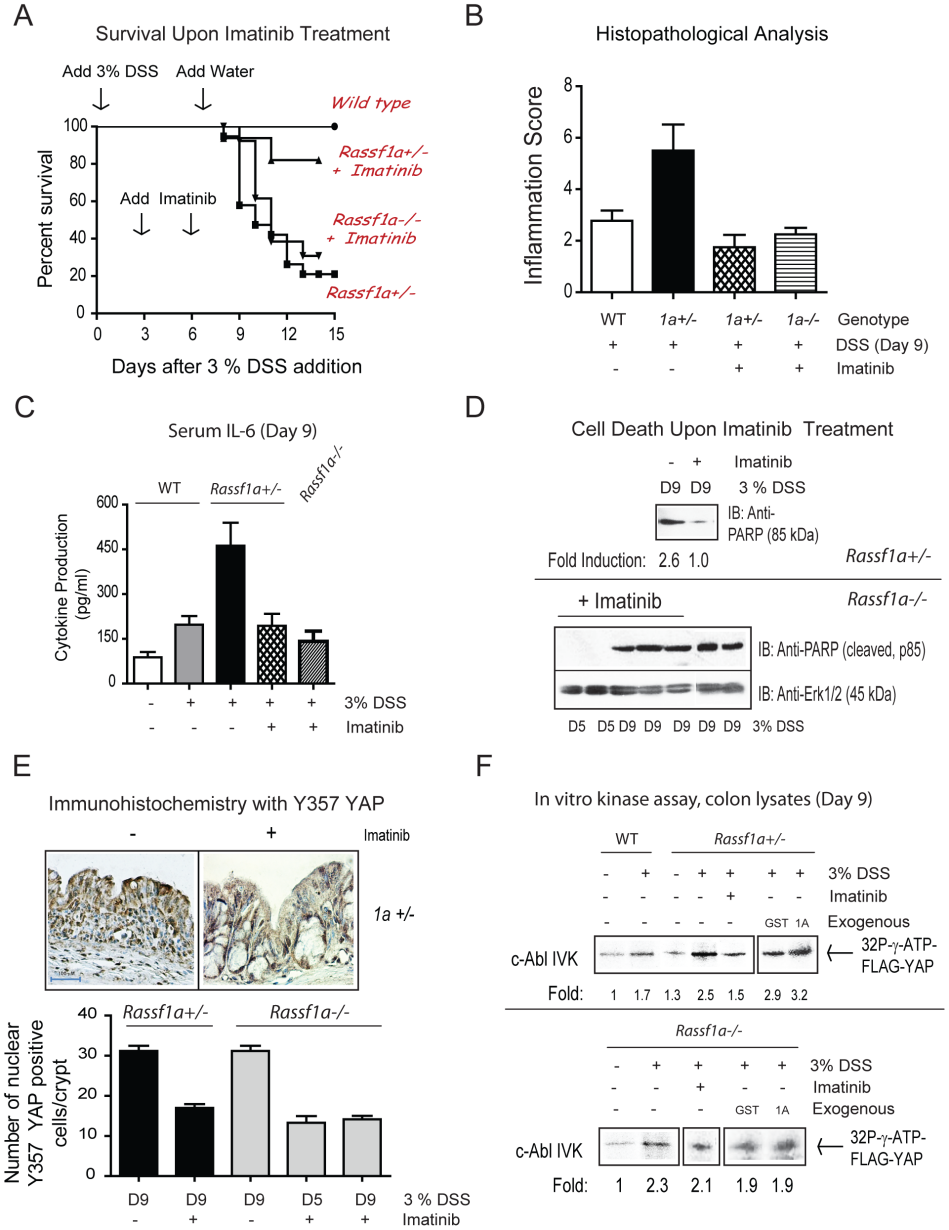
The PTK inhibitor, imatinib, inhibits the appearance of pY-YAP and promoted increased survival of *Rassf1a^+/−^* but not *Rassf1a^−/−^* mice animals following inflammation-induced injury. (A) *Rassf1a^+/−^* or *Rassf1a^−/−^* mice were intraperitoneally injected with the PTK inhibitor, imatinib at 50 mg/g body weight on days 3 and 6 following 3% DSS addition. P-value between survival of DSS-treated wild type and *Rassf1a^+/−^* was <0.0001 (n = 17) and between DSS-treated *Rassf1a^+/−^* (+ imatinib) versus DSS-treated *Rassf1a^+/−^* was 0.0086 (n = 17). No significance difference was observed between DSS-treated *Rassf1a^+/−^* and DSS-treated *Rassf1a^−/−^* (+ imatinib) mice (please see Fig. 1A for the survival curve of DSS-treated *Rassf1a^−/−^* mice). Following DSS/gleevec treatment, (B) histological analysis of colonic sections, (C) serum IL-6, (D) cell death via PARP (late marker of apoptosis); (E) phospho-YAP by IHC, and (F) *In vitro* kinase activity was carried out for c-Abl using colon lystes from DSS-treated wild type and *Rassf1a^+/−^* (top panel) and *Rassf1a^−/−^* (bottom panel) mice with overexpressed FLAG-YAP as substrate. Expression levels of c-Abl were similar in all the lanes (data not shown) and bacterially expressed GST or GST-1A (1A) was used to explore how RASSF1A may directly interfere with c-Abl kinase activity. Expression of FLAG-YAP, GST and GST-1A are shown in Fig. S7D. For (B) p-value between wild type versus *Rassf1a^+/−^* mice (+DSS) was 0.004, wild type versus *Rassf1a^+/−^* mice + DSS + gleevec) was 0.168 and wild type versus *Rassf1a^−/−^* mice (+DSS + gleevec) was 0.452 (n = 4 – 8). For (C), p-value between wild type versus *Rassf1a^+/−^* mice (+DSS) was 0.004 and wild type versus *Rassf1a^+/−^* mice (+ DSS + gleevec) was 0.347 and wild type versus *Rassf1a^−/−^* mice (+DSS + gleevec) was 0.262 (n = 4 – 8). For (E) P values of *Rassf1a^+/−^* mice or *Rassf1a^−/−^* mice (+DSS −/+ gleevec) was <0.001 (n = 10).

Next, we wanted to inhibit the protein tyrosine kinase (PTK) for YAP in order to prevent a pY-YAP/p73 driven transcription of pro-apoptotic genes (such as Bax) and possibly aid in promoting the enhanced survival of DSS-treated *Rassf1a* knockout mice. As mentioned earlier, YAP has been shown to be tyrosine phosphorylated by c-Abl in response to DNA damage. Imatinib/gleevec was developed to specifically inhibit c-Abl (IC_50_ of 25 nM) 50 and has been successful utilized to treat leukemia patients. However, it can also inhibit c-Kit and PDGF-R at 400 nM 50. Imatinib treatment of our *Rassf1a^+/−^* mice (IP injections of 60 mg/kg body weight of imatinib on day 3 and 6 of an acute 3% DSS treatment) resulted in >80% survival (Fig. 8A), low disease activity indices (Fig. S6A), low histopathological scoring of <2 (*Rassf1a^+/−^* mice normally have >6, Fig. 8B), reduced IL-6 (Fig. 8C), reduced cell death (Fig. 8D and Fig. S6B), reduced DNA and oxidative damage (Fig. S6C – D, purity of nuclear extracts is shown in S6E), regain normal crypt architecture (Fig, 8E and Fig. S7A) and increased PCNA staining (increased proliferation, Fig. S7A). Imatinib significantly reduced pY-YAP as determined by immunohistochemical staining (Fig. 8E) and immunoblotting (Fig. S7B). Furthermore, there was increased c-Abl kinase activity towards FLAG-YAP in colon lysates from the *Rassf1a^+/−^* mice that was inhibited by imatinib (Fig. 8F). The increased kinase activity of c-Abl was not inhibited by a direct modulation by RASSF1A as exogenous addition of GST-RASSF1A did not interfere with the kinase activity of c-Abl as determined by our *in vitro* method (Fig. 8F, top panel, last two lanes on the right). Thus c-Abl activity may be indirectly modulated by RASSF1A and increased c-Abl activity is most likely a result of increased DNA damage during intestinal inflammation. Taken together, our data suggested that inhibition of the c-Abl class of PTKs was effective in reversing the detrimental effects of a 3% DSS treatment in DSS-treated *Rassf1a^+/−^* mice.

In contrast to imatinib-treated *Rassf1a^+/−^* mice, imatinib-treated *Rassf1a^−/−^* mice did have different survival outcomes. We did notice that the clinical onset of disease was delayed 2 – 3 days when compared to DSS-treated *Rassf1a^+/−^* or *Rassf1a^−/−^* mice (Fig. 8A). Imatinib-treated *Rassf1a^−/−^* mice had comparable disease activity indices to imatinib-treated *Rassf1a^+/−^* mice until day 8, but by days 9 – 10, imatinib-treated *Rassf1a^−/−^* mice worsened with higher disease activity indices (Fig. S6A). Surprisingly, imatinib-treated *Rassf1a^−/−^* mice revealed reduced pY-YAP 357 (Fig. 8E and Fig. S7C), reduced inflammation scores and cytokine production (Fig. 8B and C) indicating that imatinib treatment was interfering with a key mechanism promoting early intestinal damage during the DSS treatment. However, the higher disease activity indices on days 9 – 10 and poor survival of imatinib-treated *Rassf1a^−/−^* mice may be attributed to a robust degree of cell death (Fig. 8D and Fig. S6B), DNA damage (Fig. S6C, bottom panel) and c-Abl kinase activity following DSS-induced inflammation. c-Abl kinase activity was, again, not affected by the exogenous addition of GST-RASSF1A, Fig. 8F, bottom panel). Equal amounts of FLAF-YAP were utilized as revealed by Coomassie blue staining in Fig. S7D.

**Figure 9:**
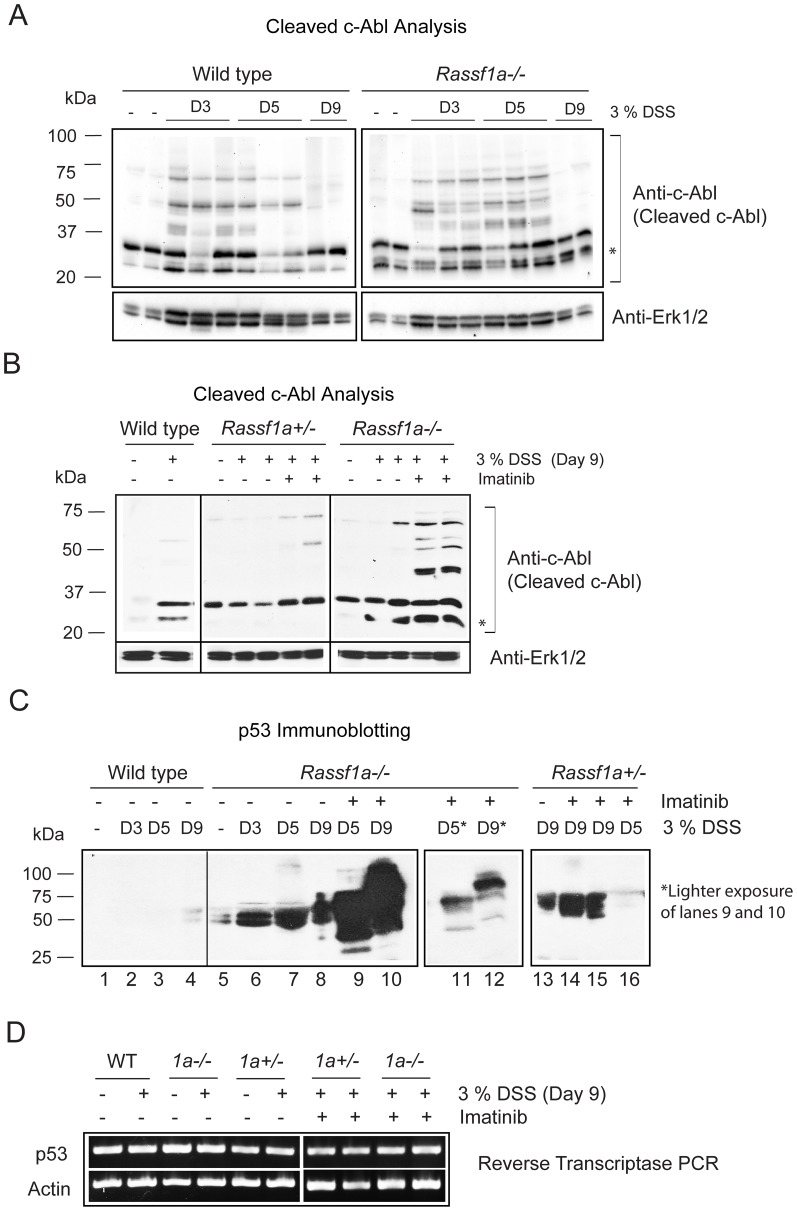
The loss of RASSF1A results in cleavage of c-Abl and accumulation of p53. (A) Expression of c-Abl following DSS treatment as indicated. Erk1/2 was used as loading control and D =  days after DSS addition. (B) Imatinib treatment of *Rassf1a^−/−^* mice (but not *Rassf1a^+/−^* mice) results in increased cleavage of c-Abl and sustained p25 cleaved form of c-Abl (indicated by *). (C) c-Abl cleavage can result in phosphorylation of Mdm2 and stabilization of p53 to promote cell death. The loss of both alleles of *Rassf1a* results in increased expression of p53 and its modification. (D) Reverse transcriptase analysis of p53 expression revealed no transcriptional changes in p53 expression following neither DSS treatment nor with imatinib addition.

Further analysis of this discrepancy, revealed that the cleavage of c-Abl was more apparent in DSS-treated *Rassf1a^−/−^* mice (Fig. 9A) and highly elevated in imatinib treated *Rassf1a^−/−^* mice (Fig. 9B), especially the presence of the predominant 25 kDa cleaved (but active) fragment of c-Abl (Fig. 9A and B, marked with “*”) 51,52. Interestingly, DSS-treated wild type animals revealed almost undetectable levels of p53 whereas DSS-treated *Rassf1a^+/−^* or *Rassf1a^−/−^* mice (Fig. 9C) revealed highly elevated or accumulated levels of p53 (as early as day 3 for the DSS-treated *Rassf1a^−/−^* mice). The accumulation of p53 was more apparent by day 9 and increased in imatinib-treated animals (Fig. 9C) whereby p53 levels not only accumulated but p53 appears to be modified to a slower migrating form around 75 kDa (Fig. 9 C, lanes 9 – 12). Under DSS or imatinib treatment, p53 mRNA levels do not appear to be significantly different, eliminating transcriptional control (Fig. 9D). Evidence for p53 stabilization can be seen in Fig. S7E and does reveal that imatinib can interfere with p53 dependent ubiquitination and increased in the DSS and imatinib-treated *Rassf1a^−/−^* mice (Fig. S7D – E). Since c-Abl can be caspase cleaved and its resultant active fragment can phosphorylate the p53 inhibitor/E3 ligase, Mdm2 53, we speculate that in the absence of RASSF1A, DSS-induced inflammation injury can result in accumulated p53 and significantly higher levels of cell death and poor recovery following DSS-induced inflammation injury.

## Discussion

**Figure 10:**
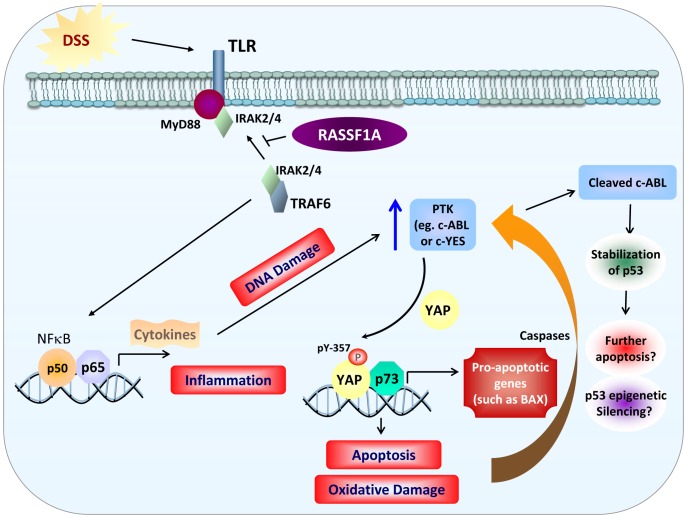
Model for RASSF1A regulation of NFκB and YAP. RASSF1A restricts NFκB activity by interfering with the ability of membrane proximal TLR/MyD88/TRAF6/IRAK2/4 to promote downstream signaling to NFκB. This results in the interference of NFκB-dependent gene transcription and the activation of inflammatory pathways. Through regulating NFκB activity (and early increases in DNA damage), RASSF1A indirectly regulates the activity (and possibly the expression) of a PTK to tyrosine phosphorylation YAP (possibly through c-Abl or c-YES). Increased PTK activity (in the absence of *Rassf1a*) would drive tyrosine phosphorylation of YAP and increased pY-YAP/p73 transcriptional up-regulation of pro-apoptotic genes such as Bax. Increased levels of Bax (and other p73/pY YAP targets) results in apoptosis, intestinal inflammation, oxidative (and DNA damage) and colonic injury. Sustained levels of apoptosis will result in the pro-apoptotic cleavage of c-Abl to stabilize p53. Accumulated p53 can further promote cell death and further colonic injury and poor recovery following inflammation insults.

An underlying hallmark of many inflammatory diseases is the enhanced activation of transcription factors, including NFκB and elevated production of NFκB -regulated cytokines. These responses are needed to promote innate immunity activation and stimulate the repair process. No single animal model can explain the pathogenesis of IBD suggesting that IBD has multifactorial etiologies 54,55. The present study argues that RASSF1A is an important regulatory element to restrict NFκB activity. Unrestricted NFκB activity can significantly contribute to poor recovery following inflammation-induced injury and inhibit the ability to promote efficient epithelial repair and survival (Fig. 10).

We have demonstrated that DSS-treated *Rassf1a^−/−^* and *Rassf1a^IEC-KO^* mice have elevated NFκB activity. Mechanistically RASSF1A appears to restrict complex formation of MyD88/TRAF6/IRAK and thus interfere with NFκB activation (Fig. 10). RASSF1A forms robust associations upon LPS or DSS stimulation with the membrane proximal complexes MyD88/TLR4 and TRAF6/IRAK2 (Fig. S8A – B) but not with TAK1, IKK or TRADD/RIP1/TNF-R1 (data not shown) in order to restrict NFκB activity. Since both LPS and DSS can stimulate TLR4, we believe that RASSF1A functions through TLR4 to inhibit IKK and the cytoplasmic/nuclear shuttling of NFκB. Indeed we have observed a significant reduction in the production of the NFκB-driven cytokine, IL-8, when RASSF1A was overexpressed in colon cancer epithelial cells (Fig. S8C). Furthermore, enhanced ubiquitination of TRAF6 can be observed in colon lysates from DSS-treated *Rassf1a^−/−^* but not wild-type mice (Fig. S8D) indicative of elevated membrane proximal activation of the TLR pathway to drive TRAF6 ubiquitination and subsequent NFκB activation (Fig. 10).

We also have *in vivo* evidence to demonstrate that YAP becomes tyrosine phosphorylated in response to DSS-induced inflammation injury most likely by the c-Abl class of PTKs. YAP was originally identified by its association with the YES Src tyrosine kinase 56 and has been demonstrated to be a transcription factor whose cytoplasmic/nuclear shuttling is controlled by post-translational phosphorylation events 57 58. During DSS-induced inflammation injury in both *Rassf1a^+/−^* and *Rassf1a^−/−^* mice, we observed elevated level of nuclear tyrosine phosphorylated YAP, enhanced YAP/p73 associations (Fig. 6E) and elevated levels of Bax expression to promote apoptosis (Fig. 6 and 7). Recently, van der Weyden et al. 59 demonstrated that the combined loss of *Rassf1a* and *Runx2* promoted the formation of oncogenic YAP1/TEAD transcriptional complexes to promote tumorigenesis. We observed reduced S127 phosphorylated YAP (possibly indicating reduced YAP/TEAD transcriptional complexes) in DSS-treated *Rassf1a^−/−^* mice. We propose that our results demonstrate that the early loss of *Rassf1a* can lead to enhanced inflammation, a key pre-condition to cancer and inflammation driven activation of YAP/p73 complexes to promote cell death. YAP1/TEAD complexes may arise after prolonged chronic inflammation to promote proliferation in support of the findings of van der Weyden et al. 59. It has been demonstrated that IL-6 can promote the activation of DNA methyltransferase 1 (DNMT1) 24,60 and DNMT1 can epigenetically silence RASSF1A 23,61 to provide a mechanism whereby inflammation may drive epigenetic silencing of RASSF1A and loss of the function of this tumor suppressor protein.

The c-Abl tyrosine kinase can phosphorylate YAP upon DNA damage, selectively promoting a YAP/p73 complex to drive the expression of pro-apoptotic genes 47. Since we cannot observe inhibition of c-Abl kinase by exogenous addition of RASSF1A, we speculate that NFκB may transcriptionally up-regulate a PTK or that NFκB dependent-inflammation will promote DNA damage to drive the tyrosine phosphorylation of YAP via c-Abl or c-Abl-like kinases (Fig. 10). Either mode of regulation would result in an abnormal tyrosine phosphorylation of YAP to yield increased epithelial apoptosis and p53 accumulation to promote further apoptosis. These events would result in poor survival following intestinal inflammation induced injury. Inhibiting this abnormal phosphorylation of YAP using the c-Abl class of PTK inhibitors (imatinib) reversed the damaging effects of DSS induced intestinal inflammation but only in a *Rassf1a^+/−^* background. In a *Rassf1a^−/−^* background, imatinib partially reversed the effects of DSS-induced inflammation but did not allow the animal to recover due a robust accumulation of p53 towards day 9 that may have resulted in more apoptosis and poor recovery. Interestingly, in day 9 imatinib-treated *Rassf1a^−/−^* mice, p53 was accumulated and modified (Fig. 9C, lane 9). We have not identified what this modification is (most likely one of many p53 phosphorylated residues) but we are currently exploring this and, if unique, may be an interesting biomarker for disease prognosis.

It is known that caspase cleaved c-Abl can phosphorylate the p53 E3 ligase, Mdm2, to inhibit Mdm2-dependent degradation of p53 and allow it to accumulate 53. Accumulated p53 can then associate with the anti-apoptotic inhibitor, Bcl-2 to promote cell death 62. This may explain why imatinib-treated *Rassf1a^−/−^* mice are not protected from DSS-induced inflammation and have similar or enhanced cell death. Curiously, we cannot absolutely eliminate tyrosine phosphorylation of YAP using imatinib (Fig. S7B) nor most of the biomarkers of inflammation or cell death. This would suggest that other PTKs may also influence tyrosine phosphorylation of YAP upon inflammation induced injury in the absence of RASSF1A. YES, the other known PTK for YAP, has two NFκB binding sites within its promoter region (as documented by http://www.genecards.org/cgi-bin/carddisp.pl?gene=YES1) and could be up-regulated by NFκB activation due to the failure of RASSF1A to restrict NFκB activation (Fig. 10). Preliminary results do reveal increased c-YES expression upon DSS-induced stimulation in *Rassf1a^+/−^* and *Rassf1a^−/−^* colon lysates (data not shown). We are currently exploring how NFκB may regulate YES expression, if YES kinase activity is elevated in DSS-treated *Rassf1a* knockout mice and if the YES inhibitor, dasatinib, can promote the survival of DSS-treated *Rassf1a* knockout mice.

Our results demonstrate that the loss of *Rassf1a* resulted in the loss of MST1 kinase activity (Fig. S5B) and the increased nuclear presence of tyrosine phosphorylation of YAP (Fig. 6F and 7A). The studies by van der Weyden et al. 59, Ren et al. 42, Cai et al. 43, Zhou et al. 63 and Matallans et al. 64 explored MST/RASSF1A dependent-regulation of YAP serine phosphorylation. However, during intestinal inflammation in our system, YAP transcriptional activities drive apoptosis in the absence of MST1/2 activity (see Fig. S5B). Recently, it was shown that YAP1 does not require MST1/2 to drive the proliferation of keratinocytes 65 in support of MST1/2 independent YAP functions. Furthermore, although Matallans et al. 64 demonstrated that RASSF1A can promote a YAP/p73 association they did not determine YAP phosphorylation linked to S127 or Y357. We argue that intestinal inflammation is driving a RASSF1A indirect affect on YAP through elevated NFκB activity to transcriptionally up-regulate a PTK to tyrosine phosphorylate YAP or, alternatively, in promoting enhanced DNA damage to activate a YAP PTK. Either way, it is an MST1/2 independent effect.

As mentioned earlier, DSS-treated *YAP^−/−^* mice have decreased survival and proliferation and increased cell death upon DSS treatment that is very similar to our inflammation phenotype 43. However, Cai et al. 43 did not explore tyrosine phosphorylation of YAP but did show reduced YAP phospho-S127 with increased time post-DSS addition 43. We have evidence that increased c-Abl cleavage (most likely by caspases) and accumulated p53 may occur in parallel to p73/YAP driven cell death following DSS-induced inflammation injury (based on the results in Fig. 9C). This may explain why DSS-treated *YAP^−/−^* mice have elevated apoptosis and poor survival in the absence of YAP. It has been shown in chronic UC patients that p53 is up-regulated in crypt cells and neutralizing antibodies to TNFα reduce the levels of p53 and IBD-related symptoms of UC patients 66 in support of the important role for p53 in intestinal epithelial-dependent cell death. In addition to p53, PUMA (p53 up-regulated modulator of apoptosis) has been shown to be up-regulated in intestinal cells following DSS treatment in mice and in colonic biopsies from UC patients suggesting an important role for other pro-apoptotic proteins in the pathogenesis of colitis 67. Preliminary microarray analyses on colonic RNA isolated from DSS-treated wild-type and *Rassf1a^−/−^* mice revealed up-regulation of several pro-apoptotic genes. These genes were increased 2 – 4 fold and include the death associated protein 3 (DAP3), programmed cell death 10 (PDCD10), PRKC apoptosis WT1 regulator (PAWR) and death effector domain-containing (DEDD) (data not shown). It will be interesting to explore if any of these are transcriptionally regulated by YAP/p73 in response to intestinal inflammation.

Efficient epithelial proliferation/repair is an integral part of recovery following inflammation-induced injury 36. Preliminary data suggest that we can observe altered β-catenin/E-cadherin adherens junctions on colon sections from DSS-treated *Rassf1a* knockout mice during the recovery phase following DSS addition (day 9, data not shown). Altered β-catenin/E-cadherin associations together with elevation of tyrosine phosphorylated YAP, will result in abnormal formation of epithelial junctions and the appearance of a “leaky gut”. Interestingly, YAP is overexpressed in CRC patients and RASSF1A is epigenetically silenced in CRC patients 12. The loss of RASSF1A may, therefore, affect other aspects of epithelial restitution with YAP activity an integral part of this process. Failure to efficiently reseal and re-establish epithelial cell homoestasis will lead to an unnecessary inflammatory response, poor recovery and an early phase of colitis-associated carcinogenesis 68,69.

An important element for recovery from inflammation-induced damage is efficient repair. During the pathogenesis of IBD, it has been documented that DNA damage 70, oxidative damage 71,72 and abnormal ROS levels 72 are elevated in a similar manner to what we have observed for DSS-treated *Rassf1a* knockout mice. Reactive oxygen species (ROS) are generated by inflammatory cells, neutrophils and macrophages and surround the inflamed mucosa 72. High levels of ROS can lead to changes in colonic membrane permeability, decreased mucosal barrier of intestinal epithelial cells and to increased DNA and oxidative damage. Both DNA and oxidative damage can lead to c-Abl activation to drive the formation of tyrosine phosphorylated YAP and a YAP/p73-dependent transcriptional program. Inhibitors of ROS generation or DNA damage may be interesting angles to pursue in the future.

In addition to c-Abl and YES as potential PTKs for YAP, preliminary microarray analyses on colonic RNA isolated from DSS-treated wild-type and *Rassf1a^−/−^* mice revealed a 7.5 fold increase in TNK2 (a tyrosine kinase, non receptor type 2 also known as ACK1) and a 2.3 fold increase in c-Mer proto-oncogene tyrosine kinase (MERTK) (data not shown). MERTK has several potential NFκB binding sites (as documented by http://www.genecards.org/cgi-bin/carddisp.pl?gene=MERTK) and can modulate the autophosphorylation of TNK2. MERTK can also regulate cytokine production and clearance of apoptotic cells. 73 Very little is known about TNK2 other than its association with cdc42 in order to maintain it in a GTP bound form 74 and TNK2-dependent tyrosine phosphorylation of the androgen and epidermal growth factor receptor 75. These observations provide interesting candidates to pursue that may function to drive the tyrosine phosphorylation of YAP and a YAP/p73 transcriptional program. Inhibiting the devastating effect of tyrosine phosphorylated YAP/p73-driven activation of pro-apoptotic genes and epithelial cell damage in the absence of RASSF1A may aid in designing future treatment schemes for patients with chronic inflammation. In fact, there has been one report to document the beneficial effects of protein tyrosine kinase inhibitors in treating IBD 26,76.

Our study provides a mechanistic understanding of DSS-induced inflammation injury in the absence of RASSF1A. Biomarker analyses for the *Rassf1a^+/−^*and *Rassf1a^−/−^* mice revealed similar outcomes suggesting similar mechanisms of disease pathogenesis (except for responsiveness to imatinib). As can be observed in Fig. 1A, we did notice a slight delay in the appearance of clinical symptoms in the *Rassf1a^+/−^* mice, especially in the appearance of rectal bleeding. However, both *Rassf1a^+/−^*and *Rassf1a^−/−^* mice had similar survival rates at the end of the experiment and the delay at the beginning of the experiment was not statistically significant. We are currently exploring biomarker analyses between day 0 and day 8 to explore molecular differences between *Rassf1a^+/−^*and *Rassf1a^−/−^* mice. Interestingly, it has recently been demonstrated that p53 functions to recruit DAXX and DNMT1 to CpG methylation sites on RASSF1A and epigenetically silence RASSF1A 77. p53 is accumulated in DSS-treated animals, in IBD patients and can also silence RASSF1A by direct promote regulation of epigenetic silencing by DNA methylation of its promoter. Furthermore, RASSF1A epigenetic silencing can be detected in ulcerative colitis patients 22 and upon IL-6 production 23. We, therefore, speculate that chronic inflammation will result in the loss of epigenetic loss of RASSF1A and RASSF1A may be in early change in the pathogenesis of ulcerative colitis. The *Rassf1a^+/−^*, *Rassf1a^−/−^* and *Rassf1a^IEC-KO^* mice may be useful models for understanding IBD and other inflammatory disorders.

## Supporting Information

Figure S1 **Genotyping and characterization of DSS-treated** ***Rassf1a^−/−^*** **mice.** (A) Crypt length of the indicated genotypes and treatments. Data was obtained from several histological sections similar to Fig. 1E. p value for the difference in crypt depth between wild type (+DSS) vs *Rassf1a* ***^−^*** ^*/****−***^ mice (+DSS)  = 0.0004 (n = 14). (B) Histological representations of the colon from DSS-treated *Rassf1a* ***^−^*** ^*/****−***^ mice at day 9. These sections further confirm the results in Fig. 1E and revealed the presence of lymphoid aggregates (dense circle of cells in top panel) in several areas of DSS treated colonic sections from *Rassf1a* ***^−^*** ^*/****−***^ mice. This would suggest active recruitment of immune cells to the inflamed area. (C) Genotyping of *Rassf1a* ***^−^*** ^*/****−***^ and *Rassf5a* ***^−^*** ^*/****−***^ animals. Immunoblot of a colon tissue preparation is shown in the bottom panel for RASSF1A and the loss of RASSF5A in the *Rassf5a* ***^−^*** ^*/****−***^ mice has been published 3. (D – E) *Rassf1a* ***^−^*** ^*/****−***^ animals show increased levels of serum or tissue myeloperoxidase (MPO) (D) and hyaluronic acid (HA, E). For Serum MPO, p-values wild type (+DSS) vs *Rassf1a* ***^−^*** ^*/****−***^ mice (+DSS) <0.0001; *Rassf1a* ***^−^*** ^*/****−***^ (+DSS) vs *Rassf5a* ***^−^*** ^*/****−***^ mice (+DSS) 0.035. Tissue MPO, p-value [wild type vs *Rassf5a* ***^−^*** ^*/****−***^ (+DSS)]  = 0.2 and (C – F) wild type (+/+) vs *Rassf5a* ***^−^*** ^*/****−***^ (treated) varied between 0.2 to 0.7 with n = 4–6. For serum HA, p-values wild type (+DSS) vs *Rassf1a* ***^−^*** ^*/****−***^ mice (+DSS) is 0.0043; *Rassf1a* ***^−^*** ^*/****−***^ (+DSS) vs *Rassf5a* ***^−^*** ^*/****−***^ mice (+DSS) 0.0099. n = 10–15 for each biomarker.
(TIF)

Figure S2 ***Ex vivo*** **analysis of BMDM and splenocytes from the indicated genotypes**. BMDM (A – D) and splenoctyes (B, D – F) were cultured, stimulated with 0.2 µg/ml LPS for 5 hours or 3% DSS for 16 – 20 hours as indicated. Supernatants were harvested and secreted cytokines quantitated by ELISA; n = 6. P-values between wild type and *Rassf1a* ***^−^*** ^*/****−***^ LPS-stimulated cells are indicated and was found to be the same for both BMDM and splenocytes in (B and D). P values between wild-type and *Rassf5a* ***^−^*** ^*/****−***^ in S2E were 0.454. For (C), p-value = 0.7 by One-way Anova analysis. n = 4–6 from 2 – 3 independent experiments for all panels. P values for the differences between *Rassf5a* ***^−^*** ^*/****−***^ versus wild-type for all panels was >0.1. For (A, C, E–F) all untreated results for *Rassf1a* ***^−^*** ^*/****−***^ and *Rassf5a* ***^−^*** ^*/****−***^ were similar to wild type (untreated). The NFκB inhibitor utilized was 10 µM methoxyresveratrol.
(EPS)

Figure S3 **Further characterization of DSS-treated intestinal cell-specific knockout** **(** ***Rassf1a^IEC-KO^*** **) animals.** (A) Crypt length of the indicated genotypes and treatments. Data was obtained from several histological sections similar to Fig. 4E. n = 15 and p-value *Rassf1a ^IEC-WT^* vs *Rassf1a ^IEC-KO^* (+DSS)  = 0.0001. (B) Tissue levels of hyaluronic acid were determined by ELISA (day 9). P-value for the difference between *Rassf1a^IEC-WT^* and *Rassf1a^IEC-KO^* (+DSS) was <0.04, n = 4. (C – E) *Ex vivo* analyses of BMDM and splenocytes from the indicated genotypes. BMDM (C) or splenocytes (D) were cultured *ex vivo* and stimulated with DSS for 16 – 20 hours or LPS for 5 hours. Supernatants were harvested and the amount of secreted cytokines quantified by ELISA; n = 4. p values *Rassf1a^IEC-WT^* vs *Rassf1a^IEC-KO^* DSS-stimulated BMDM is  = 0.8 for all cytokines in (C). p values *Rassf1a^IEC-WT^* vs *Rassf1a^IEC-KO^* LPS-stimulated splenocytes  = 0.002 (n = 8) for (D). (E) Immunoblot of colon and kidney tissue preparations from *Rassf1 ^IEC-WT^* and *Rassf1a^IEC-KO^* mice is shown. For (B–E) all untreated results for *Rassf1a^IEC-KO^* and *Rassf1a^IEC-WT^* were similar to wild type (untreated).
(EPS)

Figure S4 **Normal kidney function in** ***Rassf1a^IEC-KO^*** **animals following DSS treatment.** Serum creatinine (A) and systolic blood pressure (B) was measured as indicated (day 9 post-DSS treatment). P value was between 0.2 and 0.5 for the differences between *Rassf1a^IEC-WT^* vs *Rassf1 ^IEC-KO^* animals for these measurements (n = 8–10 for the serum creatinine levels and 6 for the blood pressure measurements). (C) Kidney sections from the indicated genotypes were analyzed after H&E staining and were found to be unaltered with and without DSS treatment.
(TIF)

Figure S5 **The loss of RASSF1A results in decreased crypt cell proliferation and increased cell death following DSS-induced inflammation injury**. (A) Measurement of PCNA positive proliferation in colonic sections. Biotinylated PCNA staining was detected using diaminobenzidine (DAB) streptavidin. HRP appears as a brown precipitated over the H&E stained sections. “*” p value  = 0.0001 and “**”  = 0.0002, n  = 12–15 for all genotypes and treatments. P value wild type (+DSS) vs *Rassf5a* ***^−^*** ^*/****−***^ (+DSS)  = 0.719. Percent PCNA staining was calculated by counting 4 groups of ∼100 cells in three independent histological sections from each genotype and treatment. (B) MST1 *in vitro* kinase assay was carried out on colon lysates from the indicated genotypes using Histone H2B. MST1 expression is shown for the −/+ DSS-treated *Rassf1a* ***^−^*** ^*/****−***^ mice. Similar results were obtained for the other genotypes and experiment was carried out twice with similar results. (C) Time course analysis of DNA damage utilizing expression of phospho-γ-H2AX as observed in Fig. 7C. (D) Fluorometric analysis of production of reactive oxygen species (ROS) using intracellular oxidation of 2′,7′-dichlorofluorescin diacetate (DCF-DA) by freshly isolated colon crypt cells from the indicated genotypes. P values wild-type (+/+, +DSS) vs *Rassf1a* ***^−^*** ^*/****−***^ or *Rassf1a^+/^* ^***−***^ mice (+DSS) <0.0001; wild type (+DSS) vs *Rassf1a^IEC-KO^* mice  = 0.0006; wild type (+DSS) vs *Rassf5a* ***^−^*** ^*/****−***^ mice (+DSS) 0.083 (n = 8 for all genotypes). For (A and C) all untreated results for *Rassf1a* ***^−^*** ^*/****−***^, *Rassf5a* ***^−^*** ^*/****−***^, *Rassf1a^IEC-KO^* were similar to wild type (untreated).
(TIF)

Figure S6 **The PTK inhibitor, imatinib reverses the damaging effects of DSS treatment in the** ***Rassf1a^+/−^*** **but not** ***Rassf1a^−/−^*** **knockout mice**. Imatinib was administered intraperitoneally at 50 mg/g body weight on day 3 and 6 and (A) Disease activity index, (B) cell death using Bax immunoblotting (as an early marker of apoptosis) (in colon lysates), (C) the DNA damage marker phospho-γ-H2AX (in colon lysates) and (D) the oxidative damage marker, HO-1was carried out as indicated (source of sample was colonic mRNA). (E) Purity of our nuclear and cytoplasmic fractions was tested as indicated.
(EPS)

Figure S7 **Further analysis of biomarkers of intestinal inflammation were analyzed**. (A) PCNA staining with quantitation on the right panel, (B) Detection of pY-YAP was carried out as indicated, (C) pY-YAP immunohistochemistry carried out, (D) Expression of FLAG-YAP (top panel), GST and GST-1A (bottom panel) used in *in vitro* kinase assay in Fig. 8F.(E) Ubiqutination of p53 was carried out as indicated in colon lysate samples. All baseline (untreated) results not shown were significantly not different from wild type (untreated).
(TIF)

Figure S8 **RASSF1A restricts NF**κ**B signaling by association with membrane proximal complexes.** (A – B) HT-29 (a colon epithelial cancer) cells were transiently transfected with vector (-) or HA-tagged RASSF1A (HA-1A). Fourty-eight hours after transfection, cells were grown in serum free media for two hours followed by (A) 1 µg/ml LPS addition or (B) 3% DSS in the media, lysed and immunoprecipitated with the indicated antibodies. Associated proteins were separated on SDS-PAGE and immunoblotted. (C) HT-29 (colon epithelial cancer) cells were transiently transfected with vector (-) or HA-tagged RASSF1A (HA-1A). Fourty-eight hours after transfection, cells were grown in serum free media for two hours followed by the addition of 1 µg/ml LPS or 3% DSS for 5 hours. One mL of the supernatant was harvested and IL-8 levels determined. Inset, expression of HA-1A in HT-29 cells. Similar results were obtained for HCT-116 cells (data not shown). (D) Colon extracts were prepared from the indicated genotypes −/+DSS treated, immunoprecipitated with anti-TRAF6 and immunoblotted as indicated. Lysates from two animals/treatement/genotype were analyzed as indicated.
(EPS)

Methods S1 **Supplementary Methods Text S1.**
(DOCX)
