# Correction: The Tumor Suppressor Gene, RASSF1A, Is Essential for Protection against Inflammation -Induced Injury

**DOI:** 10.1371/annotation/0ed852a0-eb39-4a28-986f-81f28d870747

**Published:** 2014-01-06

**Authors:** Marilyn Gordon, Mohamed El-Kalla, Yuewen Zhao, Yahya Fiteih, Jennifer Law, Natalia Volodko, Anwar Anwar-Mohamed, Ayman O. S. El-Kadi, Lei Liu, Jeff Odenbach, Aducio Thiesen, Christina Onyskiw, Haya Abu Ghazaleh, Jikyoung Park, Sean Bong Lee, Victor C. Yu, Carlos Fernandez-Patron, R. Todd Alexander, Eytan Wine, Shairaz Baksh

The name of the seventh author is incorrect. The correct name is Anwar Anwar-Mohamed. The abbreviation of this name in the Author Contributions Statement is correct as it is. 

