# Correction: The Tumor Suppressor Gene, RASSF1A, Is Essential for Protection against Inflammation -Induced Injury

**DOI:** 10.1371/journal.pone.0118034

**Published:** 2015-02-02

**Authors:** The PLOS ONE Staff

There is an error in the legend for Fig. 8. Please see the corrected Fig. 8 here.

**Figure 8:**
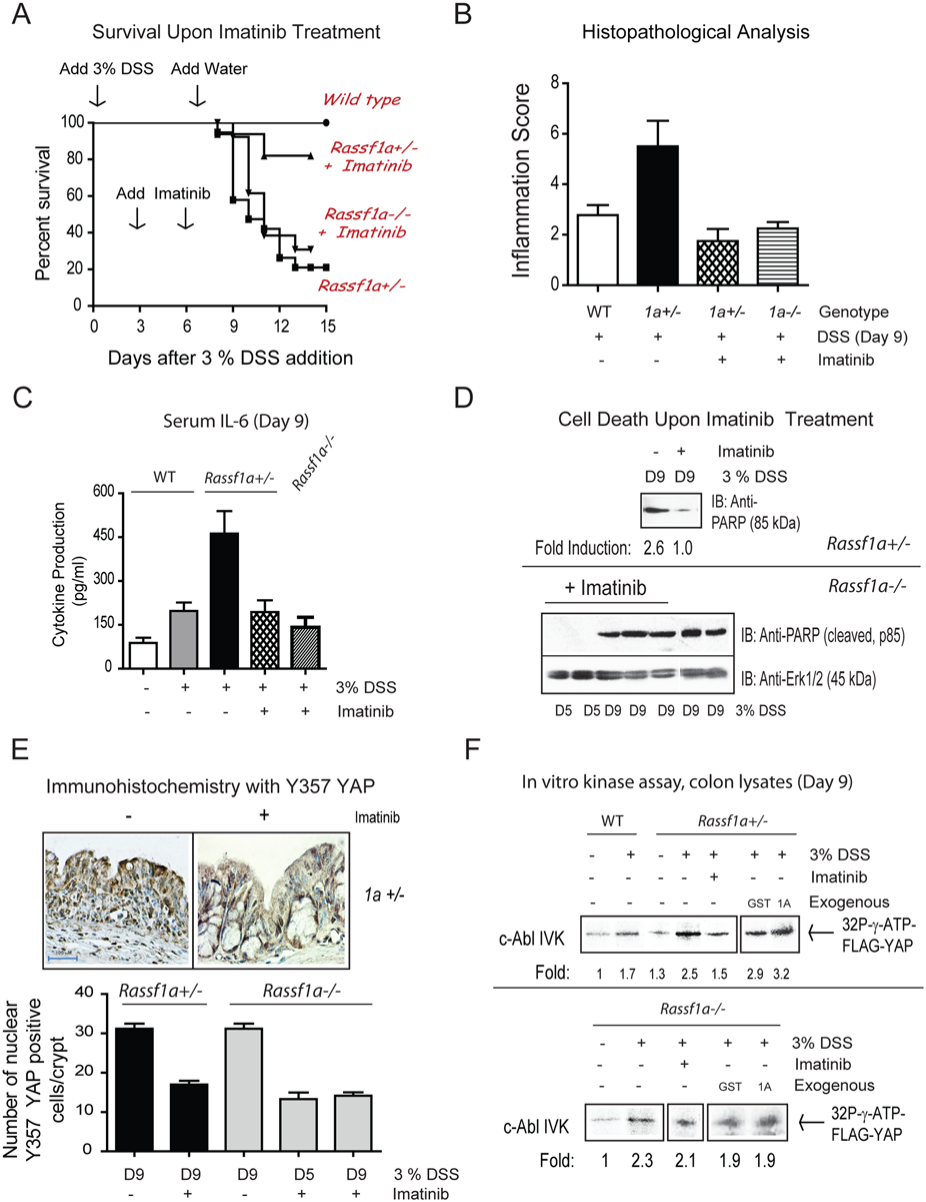
The PTK inhibitor, imatinib, inhibits the appearance of pY-YAP and promoted increased survival of *Rassf1a^+/−^* but not *Rassf1a^−/−^* mice animals following inflammation-induced injury. (A) *Rassf1a^+/−^* or *Rassf1a^−/−^* mice were intraperitoneally injected with the PTK inhibitor, imatinib at 60 mg/kg body weight on days 3 and 6 following 3% DSS addition. P-value between survival of DSS-treated wild type and *Rassf1a^+/−^* was <0.0001 (n = 17) and between DSS-treated *Rassf1a^+/−^* (+ imatinib) versus DSS-treated *Rassf1a^+/−^* was 0.0086 (n = 17). No significance difference was observed between DSS-treated *Rassf1a^+/−^* and DSS-treated *Rassf1a^−/−^* (+ imatinib) mice (please see Fig. 1A for the survival curve of DSS-treated *Rassf1a^−/−^* mice). Following DSS/gleevec treatment, (B) histological analysis of colonic sections, (C) serum IL-6, (D) cell death via PARP (late marker of apoptosis); (E) phospho-YAP by IHC, and (F) *In vitro* kinase activity was carried out for c-Abl using colon lystes from DSS-treated wild type and *Rassf1a^+/−^* (top panel) and *Rassf1a^−/−^* (bottom panel) mice with overexpressed FLAG-YAP as substrate. Expression levels of c-Abl were similar in all the lanes (data not shown) and bacterially expressed GST or GST-1A (1A) was used to explore how RASSF1A may directly interfere with c-Abl kinase activity. Expression of FLAG-YAP, GST and GST-1A are shown in Fig. S7D. For (B) p-value between wild type versus *Rassf1a^+/−^* mice (+DSS) was 0.004, wild type versus *Rassf1a^+/−^* mice + DSS + gleevec) was 0.168 and wild type versus *Rassf1a^−/−^* mice (+DSS + gleevec) was 0.452 (n = 4 – 8). For (C), p-value between wild type versus *Rassf1a^+/−^* mice (+DSS) was 0.004 and wild type versus *Rassf1a^+/−^* mice (+ DSS + gleevec) was 0.347 and wild type versus*Rassf1a^−/−^* mice (+DSS + gleevec) was 0.262 (n = 4 – 8). For (E) P values of *Rassf1a^+/−^* mice or*Rassf1a^−/−^* mice (+DSS −/+ gleevec) was <0.001 (n = 10).
