# Correction: The Tumor Suppressor Gene, RASSF1A, Is Essential for Protection against Inflammation -Induced Injury

**DOI:** 10.1371/journal.pone.0131150

**Published:** 2015-06-24

**Authors:** Marilyn Gordon, Mohamed El-Kalla, Yuewen Zhao, Yahya Fiteih, Jennifer Law, Natalia Volodko, Anwar Mohamed, Ayman O. S. El-Kadi, Lei Liu, Jeff Odenbach, Aducio Thiesen, Christina Onyskiw, Haya Abu Ghazaleh, Jikyoung Park, Sean Bong Lee, Victor C. Yu, Carlos Fernandez-Patron, R. Todd Alexander, Eytan Wine, Shairaz Baksh

There is an error in the legend for S6 Fig. Please see the corrected S6 Fig. below.

There are multiple errors in the image for S7 Fig. Please see the corrected S7 Fig. below.

## Supporting Information

S6 Fig The PTK inhibitor, imatinib reverses the damaging effects of DSS treatment in the *Rassf1a^+/−^* but not *Rassf1a^−/−^* knockout mice. Imatinib was administered intraperitoneally at 60 mg/kg body weight on day 3 and 6 and (A) Disease activity index, (B) cell death using Bax immunoblotting (as an early marker of apoptosis) (in colon lysates), (C) the DNA damage marker phospho-γ-H2AX (in colon lysates) and (D) the oxidative damage marker, HO-1was carried out as indicated (source of sample was colonic mRNA). (E) Purity of our nuclear and cytoplasmic fractions was tested as indicated.
(TIF)

S7 Fig Further analysis of biomarkers of intestinal inflammation were analyzed. (A) PCNA staining with quantitation on the right panel, (B) Detection of pY-YAP was carried out as indicated, (C) pY-YAP immunohistochemistry carried out, (D) Expression of FLAG-YAP (top panel), GST and GST-1A (bottom panel) used in *in vitro* kinase assay in Fig. 8F.(E) Ubiqutination of p53 was carried out as indicated in colon lysate samples. All baseline (untreated) results not shown were significantly not different from wild type (untreated).
(PDF)
