# Supplementary figures and images for: The Tumor Suppressor Gene, RASSF1A, Is Essential for Protection against Inflammation -Induced Injury
Source: PLoS One. 2013 Oct 16;8(10):e75483. doi: 10.1371/journal.pone.0075483 (PMC3797720; doi:10.1371/journal.pone.0075483)

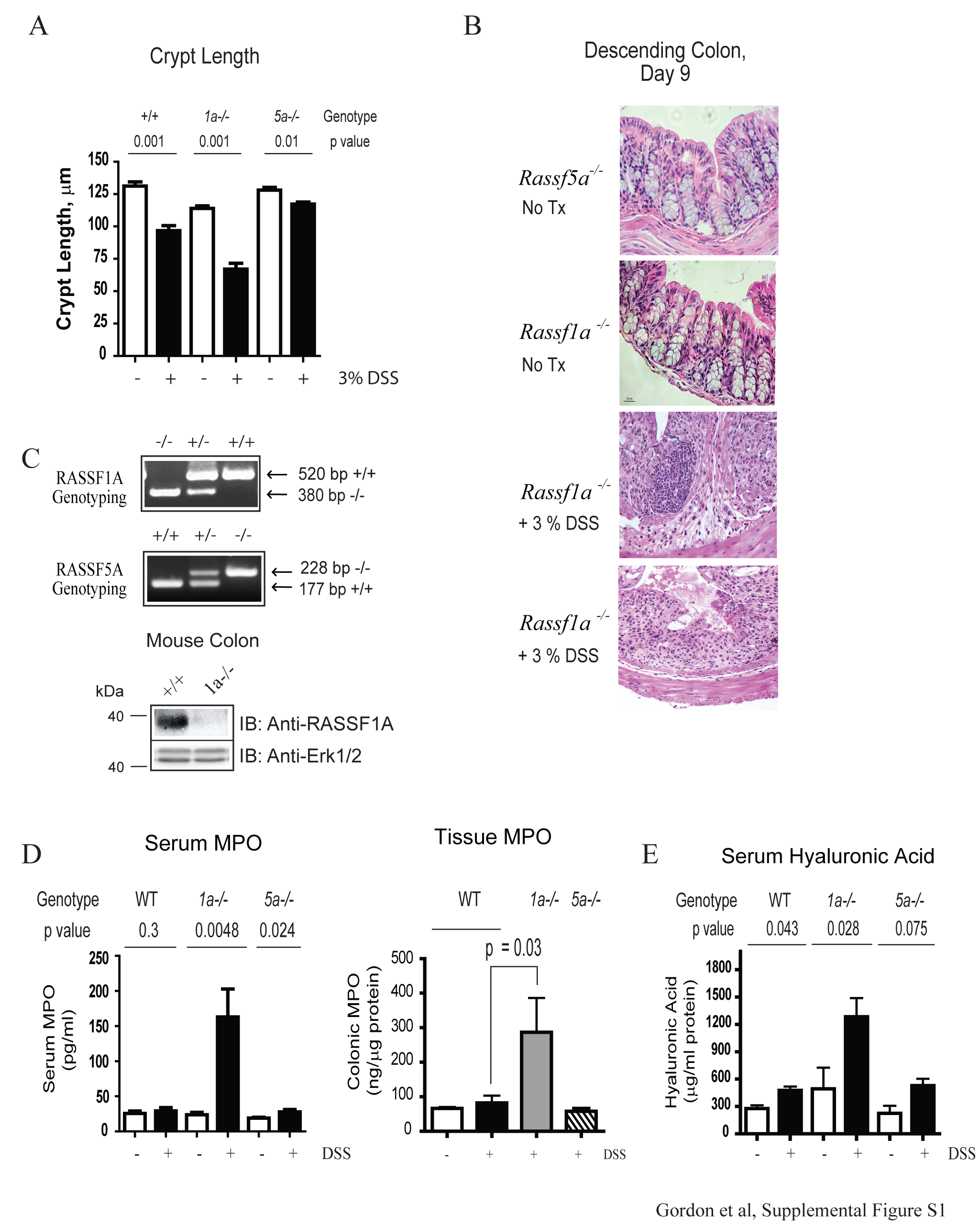

Supplement: Figure S1 — Genotyping and characterization of DSS-treated Rassf1a−/− mice. (A) Crypt length of the indicated genotypes and treatments. Data was obtained from several histological sections similar to Fig. 1E. p value for the difference in crypt depth between wild type (+DSS) vs Rassf1a − /− mice (+DSS) = 0.0004 (n = 14). (B) Histological representations of the colon from DSS-treated Rassf1a − /− mice at day 9. These sections further confirm the results in Fig. 1E and revealed the presence of lymphoid aggregates (dense circle of cells in top panel) in several areas of DSS treated colonic sections from Rassf1a − /− mice. This would suggest active recruitment of immune cells to the inflamed area. (C) Genotyping of Rassf1a − /− and Rassf5a − /− animals. Immunoblot of a colon tissue preparation is shown in the bottom panel for RASSF1A and the loss of RASSF5A in the Rassf5a − /− mice has been published 3. (D – E) Rassf1a − /− animals show increased levels of serum or tissue myeloperoxidase (MPO) (D) and hyaluronic acid (HA, E). For Serum MPO, p-values wild type (+DSS) vs Rassf1a − /− mice (+DSS) <0.0001; Rassf1a − /− (+DSS) vs Rassf5a − /− mice (+DSS) 0.035. Tissue MPO, p-value [wild type vs Rassf5a − /− (+DSS)] = 0.2 and (C – F) wild type (+/+) vs Rassf5a − /− (treated) varied between 0.2 to 0.7 with n = 4–6. For serum HA, p-values wild type (+DSS) vs Rassf1a − /− mice (+DSS) is 0.0043; Rassf1a − /− (+DSS) vs Rassf5a − /− mice (+DSS) 0.0099. n = 10–15 for each biomarker. (TIF) [file pone.0075483.s001.tif]

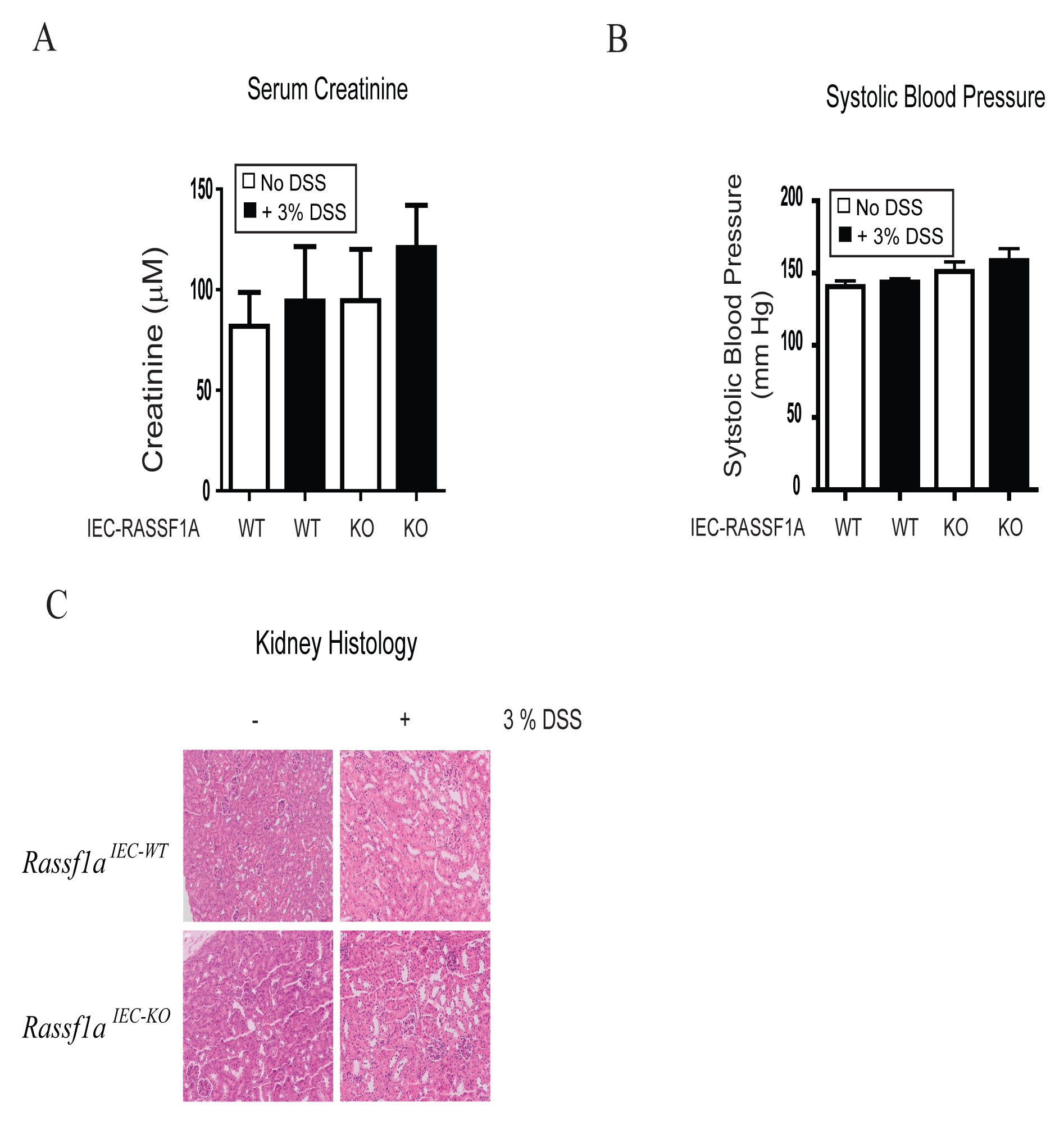

Supplement: Figure S4 — Normal kidney function in Rassf1aIEC-KO animals following DSS treatment. Serum creatinine (A) and systolic blood pressure (B) was measured as indicated (day 9 post-DSS treatment). P value was between 0.2 and 0.5 for the differences between Rassf1aIEC-WT vs Rassf1 IEC-KO animals for these measurements (n = 8–10 for the serum creatinine levels and 6 for the blood pressure measurements). (C) Kidney sections from the indicated genotypes were analyzed after H&E staining and were found to be unaltered with and without DSS treatment. (TIF) [file pone.0075483.s004.tif]

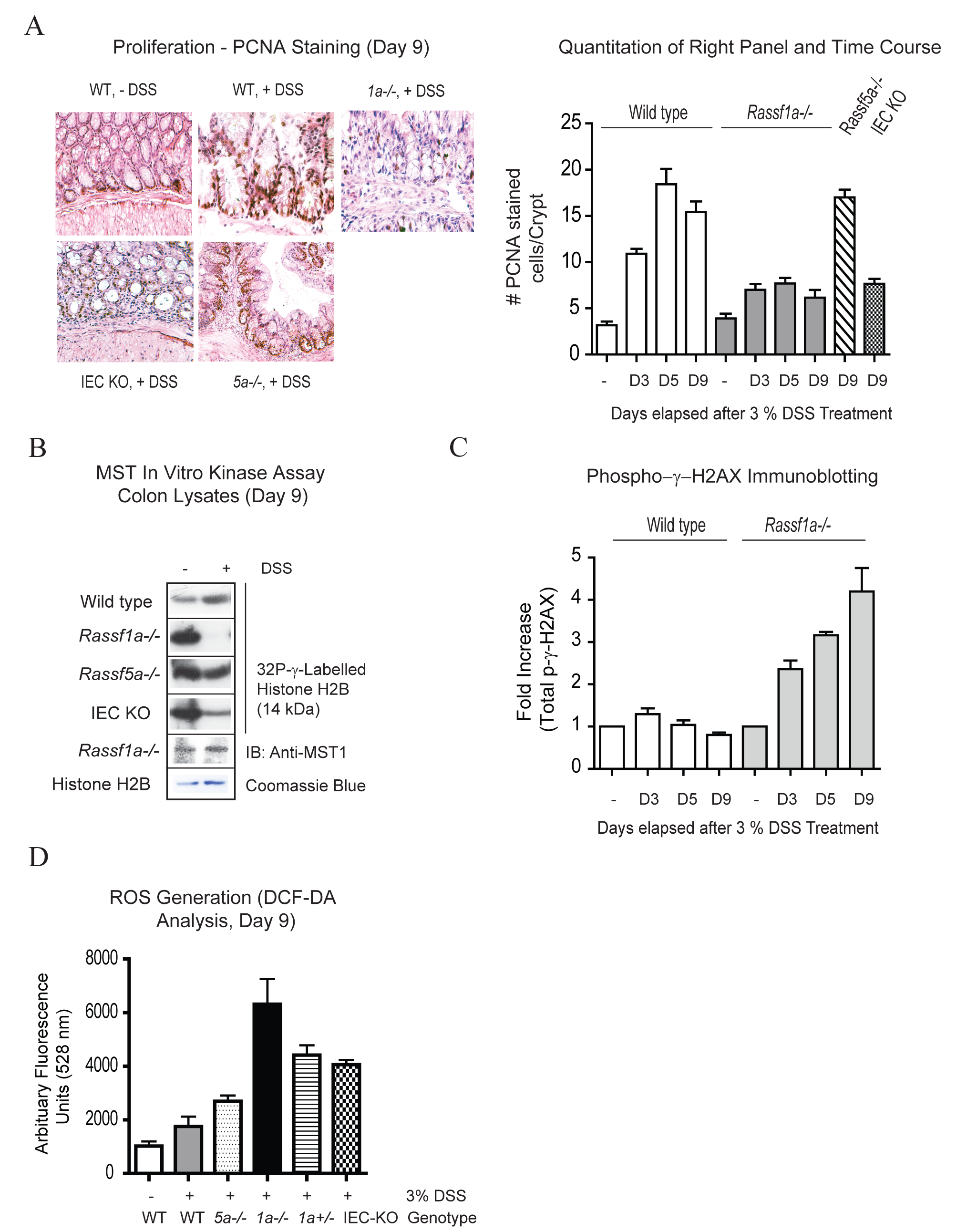

Supplement: Figure S5 — The loss of RASSF1A results in decreased crypt cell proliferation and increased cell death following DSS-induced inflammation injury. (A) Measurement of PCNA positive proliferation in colonic sections. Biotinylated PCNA staining was detected using diaminobenzidine (DAB) streptavidin. HRP appears as a brown precipitated over the H&E stained sections. “*” p value = 0.0001 and “**” = 0.0002, n = 12–15 for all genotypes and treatments. P value wild type (+DSS) vs Rassf5a − /− (+DSS) = 0.719. Percent PCNA staining was calculated by counting 4 groups of ∼100 cells in three independent histological sections from each genotype and treatment. (B) MST1 in vitro kinase assay was carried out on colon lysates from the indicated genotypes using Histone H2B. MST1 expression is shown for the −/+ DSS-treated Rassf1a − /− mice. Similar results were obtained for the other genotypes and experiment was carried out twice with similar results. (C) Time course analysis of DNA damage utilizing expression of phospho-γ-H2AX as observed in Fig. 7C. (D) Fluorometric analysis of production of reactive oxygen species (ROS) using intracellular oxidation of 2′,7′-dichlorofluorescin diacetate (DCF-DA) by freshly isolated colon crypt cells from the indicated genotypes. P values wild-type (+/+, +DSS) vs Rassf1a − /− or Rassf1a+/ − mice (+DSS) <0.0001; wild type (+DSS) vs Rassf1aIEC-KO mice = 0.0006; wild type (+DSS) vs Rassf5a − /− mice (+DSS) 0.083 (n = 8 for all genotypes). For (A and C) all untreated results for Rassf1a − /−, Rassf5a − /−, Rassf1aIEC-KO were similar to wild type (untreated). (TIF) [file pone.0075483.s005.tif]

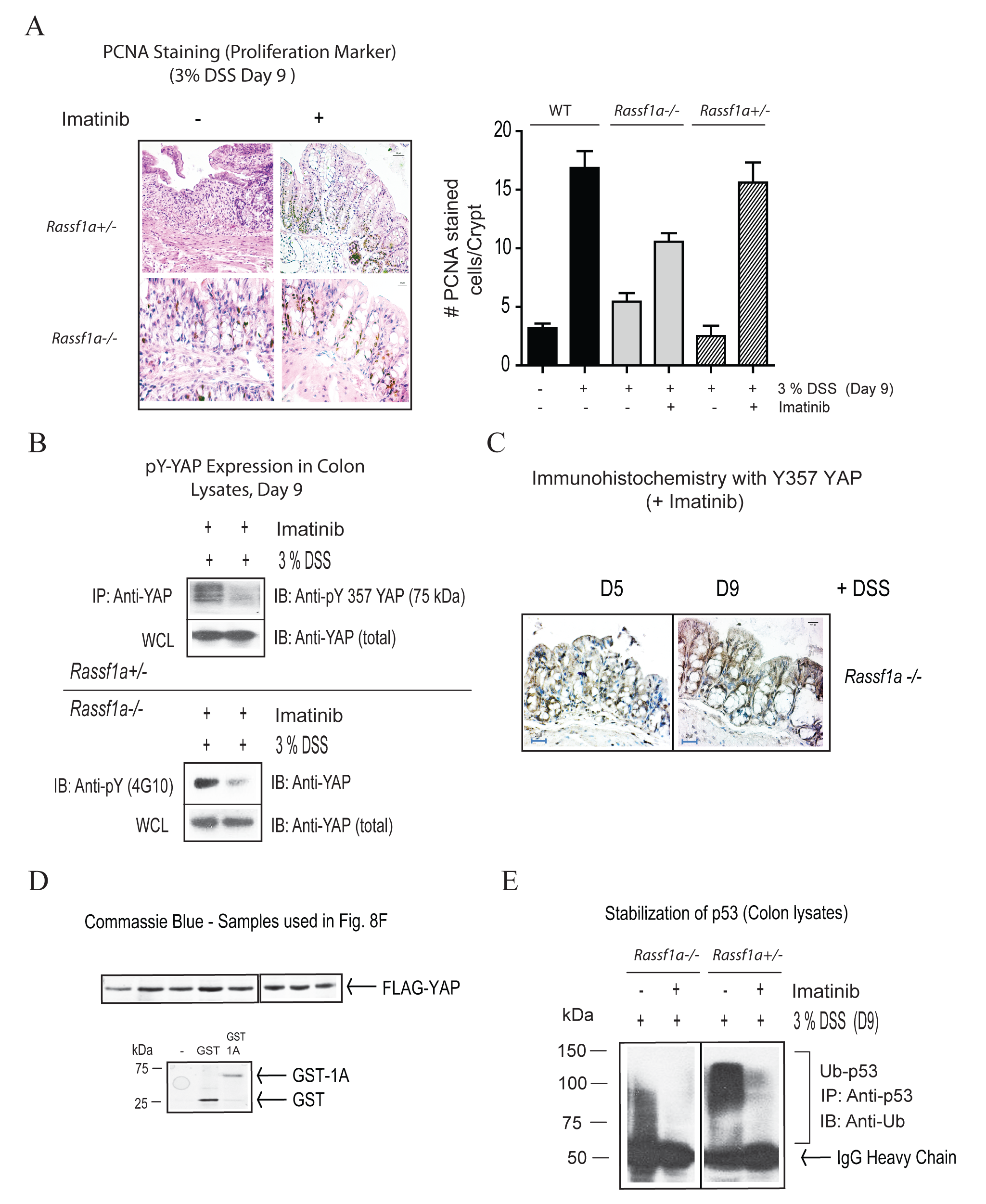

Supplement: Figure S7 — Further analysis of biomarkers of intestinal inflammation were analyzed. (A) PCNA staining with quantitation on the right panel, (B) Detection of pY-YAP was carried out as indicated, (C) pY-YAP immunohistochemistry carried out, (D) Expression of FLAG-YAP (top panel), GST and GST-1A (bottom panel) used in in vitro kinase assay in Fig. 8F.(E) Ubiqutination of p53 was carried out as indicated in colon lysate samples. All baseline (untreated) results not shown were significantly not different from wild type (untreated). (TIF) [file pone.0075483.s007.tif]
