# Supplementary material for: The Tumor Suppressor Gene, RASSF1A, Is Essential for Protection against Inflammation -Induced Injury
Source: PLoS One. 2013 Oct 16;8(10):e75483. doi: 10.1371/journal.pone.0075483 (PMC3797720; doi:10.1371/journal.pone.0075483)
Supplement: Methods S1 — Supplementary Methods Text S1. (DOCX) [file pone.0075483.s009.docx]

**The tumor suppressor gene, RASSF1A, is essential for protection against inflammation-induced injury**

Marilyn Gordon^1,*^, Mohamed El-Kalla^1,*^, Yuewen Zhao^2, 3, *^, Yahya Fiteih^1^, Jennifer Law^2^, Natalia Volodko^1^, Anwar Mohamed^4^, Ayman O. S. El-Kadi^4^, Lei Liu^5^, Jeff Odenbach^2^, Aducio Thiesen^6^, Christina Onyskiw^1^, Haya Abu Ghazaleh^1,7^, Jikyoung Park^8,9^, Sean Bong Lee^9^, Victor C. Yu^10^, Carlos Fernandez-Patron^2^, R. Todd Alexander^1, 11^, Eytan Wine^1,5,12^ and Shairaz Baksh^1,2,12,13,14^

____________________________________________________________________________________

**Supplementary Methods Text S1**

**Antibodies and reagents**

Antibodies: Rabbit anti-Erk1/Erk2 (sc-93/sc-154), anti-IRAK2 (sc-23650), anti-Traf6 (sc-7221), anti-TLR4 (sc-10741) anti-MyD88 (sc-11356), anti-p73 (sc-7957), p53 (AbCAM), anti-c-Abl (sc-131 and BD#554148) and mouse anti-Ub (sc-8017) were purchased from Santz Cruz Biotechnology. Murine monoclonal anti-HA (12CA5) was an in house hybridoma; phospho (p)-YAP S127 (cell signaling 4911S)/pY-YAP 357 (Sigma Y4645)/total YAP (Santa Cruz sc-15407), 4G10 (Millipore 4G10 Platinum 05-1050), p-IκBα (# 9246)/total IκBα (# 9242) were from Cell signaling; anti-RASSF1A (M304 from Dr. Gerd Pfiefer). ECL detection (GE Amersham ECL RPN2106) was used for all immunostaining analysis. 2′,7′-Dichlorofluorescin diacetate, thiobarbituric acid, butylated hydroxytoluene, diethylenetriamine penta acetic acid, and diethylthiocarbamic acid were purchased from Sigma Aldrich. (St. Louis, MO). SYBR Green SuperMix, and 96-well optical reaction plates with optical adhesive films were purchased from Applied Biosystems (Foster City, CA). Real-time PCR primers were synthesized by Integrated DNA Technologies Inc. Imatinib mesylate/gleevec was obtained from Selleck Chemicals.

**Inflammation Biomarkers Analyses**

Cytokine levels were assessed in diluted serum samples or *ex vivo* cellular supernatant extracts using ELISA and carried out in accordance to manufacturer’s instructions (Pierce/Endogen). Myeloperoxidase (MPO) and hyaluronic acid (HA) assays were also carried out by ELISA (Hycult and Echelon Biosciences respectively). Crude measurements of tissue MPO activity were determined by utilizing 25 µg of proteins and tetramethylbenzidine (TMB) as substrate. The oxidation product of TMB, generated by enzymatic degradation of H_2_O_2_ by MPO, was quantified using a spectrophotometer at an absorbance of 450nm.

**Blood pressure and serum creatinine levels**

Blood pressure measurements were carried out using a computerized tail cuff plethysmography (Kent Scientific Corporatinon) [[81](#_ENREF_81)]. Briefly, conscious mice were maintained at 32 – 35 ^o^C using a heating pad and restrained during all blood pressure measurements. Averages of 10 inflation/deflation cycles were conducted to obtain mean cytosolic blood pressure. Animals were allowed to stabilize after transport before blood pressure measurements were taken. Measurements were taken at the same time of day for all the genotypes. Serum collected from DSS-treated animals were used to determine serum creatinine levels via a picrate based colorometric assay. Specifically we employed the Creatinine Parameter Assay Kit (Cat no.: KGE005, R&D Systems, Inc., Minneapolis, MN, USA).

**Cells lines and transfection**

HT-29 or HCT116 cells were maintained in McCoy’s 5A medium plus 10% bovine growth serum (BGS). HCT116 (p53^+/+^Bax^+/-^) cells were a generous gift from Bert Vogelstein. HT-29 (p53^mut^Bax^+/+^) were obtained from Dr. Eytan Wine (Department of Pediatrics, University of Alberta) [[82](#_ENREF_82)]. All cells were maintained in a 37^o^C/5% CO_2_ incubator. Transfections were carried out using the linear 25 kDa polymer, polyethyleneimine (PEI) obtained from Polysciences, USA (Catalog #23966-2). PEI transfections were carried out by mixing PEI/DNA in a ratio of 4 μL PEI/1 μg DNA in 400 μL of serum-free DMEM (for transfection in a 6 well dish) as described elsewhere [[83](#_ENREF_83)]. Further details are available from S.B. upon request.

**Immunoprecipitation, immunoblotting and immunohistochemistry**

Protein associations were carried out in lysis buffer containing 50 mM HEPES (pH 7.5), 150 mM NaCl, 1 mM MgCl_2_, 1.5 mM EDTA, 0.5% Triton X-100, 20 mM β-glycerolphosphate, 100 mM NaF, 0.1 mM PMSF followed by immunoprecipitation (IP) with 1 – 2 μg of the indicated antibodies and 500 mg of protein. Associated proteins were separated out by SDS-PAGE, transfered to PVDF membrane, and immunoblotting (IB) as indicated and as described previously. [[83](#_ENREF_83)] For all whole cell lysate (WCL) immunoblots, 10% of input was used (~ 70 μg of protein/lane). For immunohistochemistry, formalin fixed, paraffin embedded sections were deparafinized and re-hydrated as described previously [[84](#_ENREF_84)]. Antigen retrieval was done by boiling in sodium citrate buffer as previously described. Endogenous peroxidase activity was quenched with 3% H_2_O_2_. Sections were blocked in 2% BSA + donkey serum for one hour at room temperature, and incubated in 1:100 primary antibody as indicated (pY357 YAP- SigmaY4645, Bax 5B7- Trevigen 2280-MC-100) overnight at 4^o^ C. Sections were incubated in 1:500 biotinylated secondary antibody for 1 hour at room temperature (Jackson Immunoresearch 711-065-152) and signal amplification and detection was done using the VECTASTAIN Elite ABC Kit (Vector Labs PK-6100) and the Metal Enhanced DAB Substrate Kit (Pierce 34065). Counterstaining was done using Harris' modified hematoxylin (Fisher SH26-500D).

**Quantification by Real-Time PCR for HO-1**

Quantitative analysis of specific mRNA expression was performed using real-time PCR by subjecting the resulting cDNA to PCR amplification using 96-well optical reaction plates in the ABI Prism 7500 System (Applied Biosystems). The 25 μL reaction mix contained 0.1 μL of 10 μM forward primer and 0.1 μL of 10 μM reverse primer (40 nM final concentration of each primer) (Table. 1), 12.5 μL of SYBR Green Universal Mastermix, 11.05 μL of nuclease-free water, and 1.25 μL of cDNA sample. Assay controls were incorporated onto the same plate, namely, no-template controls to test for the contamination of any assay reagents. After sealing the plate with an optical adhesive cover, the thermocycling conditions were initiated at 95°C for 10 min, followed by 40 PCR cycles of denaturation at 95°C for 15 s and annealing/extension at 60°C for 1 min. Melting curve (dissociation stage) was performed by the end of each cycle to ascertain the specificity of the primers and the purity of the final PCR product.

**Real-Time PCR Data Analysis for HO-1**

The real time-PCR data were analyzed using the relative gene expression i.e. (ΔΔC_T_) method as described in Applied Biosystems User Bulletin No.2 and explained further by Livak and Schmittgen (2001).[[85](#_ENREF_85)] Briefly, the ΔC_T_ values were calculated in every sample for each gene of interest as follows: C_T gene of interest_ – C_T_ _reporter gene_, with β-actin as the reporter gene. Calculation of relative changes in the expression level of one specific gene (ΔΔC_T_) was performed by subtraction of ΔC_T_ of control (vehicle treated animals at 6 or 24 h time points) from the ΔC_T_ of the corresponding treatment groups. The values and ranges given in different figures were determined as follows: 2^–Δ(ΔCT)^ with ΔΔC_T_ + S.E. and ΔΔC_T_ – S.E., where S.E. is the standard error of the mean of the Δ(ΔC_T_) value.

**Reverse transcriptase PCR for p53 and actin**

A total of 2 μg of total RNA was converted into cDNA with an Applied Biosystems high-capacity cDNA Reverse Transcription Kit according to manufacturer's instructions. After reverse transcription cDNA was diluted 10 times with RNase-free water and 5 μL was used in PCR reactions using NEB Taq DNA polymerase with standard Taq buffer (<https://www.neb.com/products/m0273-taq-dna-polymerase-with-standard-taq-buffer>). PCR parameters were denaturation 94 ^o^C for 2 min (1 cycle), 94 ^o^C for 1 min, 60 ^o^C or 55 ^o^C (for p53 and actin PCR respectively) for 1 min and 68 ^o^C for 1 min (35 cycles) followed by a final extension 68 ^o^C for 10 min. PCR products were analyzed on a 2 % agarose gel and visualized with ethidium bromide. The following primers were used: beta-actin (product of 165 bp) forward primer was 5'-GTGACGTTGACATCCGTAAAGA-3’ and reverse primer was 5'-GCCGGACTCATCGTACTCC-3'. For p53 (product size of 182 bp), forward primer was 5'-TCAGTTCATTGGGACCATCCTG-3' and reverse primer was 5'-AAAATGTCTCCTGGCTCAGAGG-3'.
