# Supplementary figures and images for: Correction: The Tumor Suppressor Gene, RASSF1A, Is Essential for Protection against Inflammation -Induced Injury
Source: PLoS One. 2015 Jun 24;10(6):e0131150. doi: 10.1371/journal.pone.0131150 (PMC4479583; doi:10.1371/journal.pone.0131150)

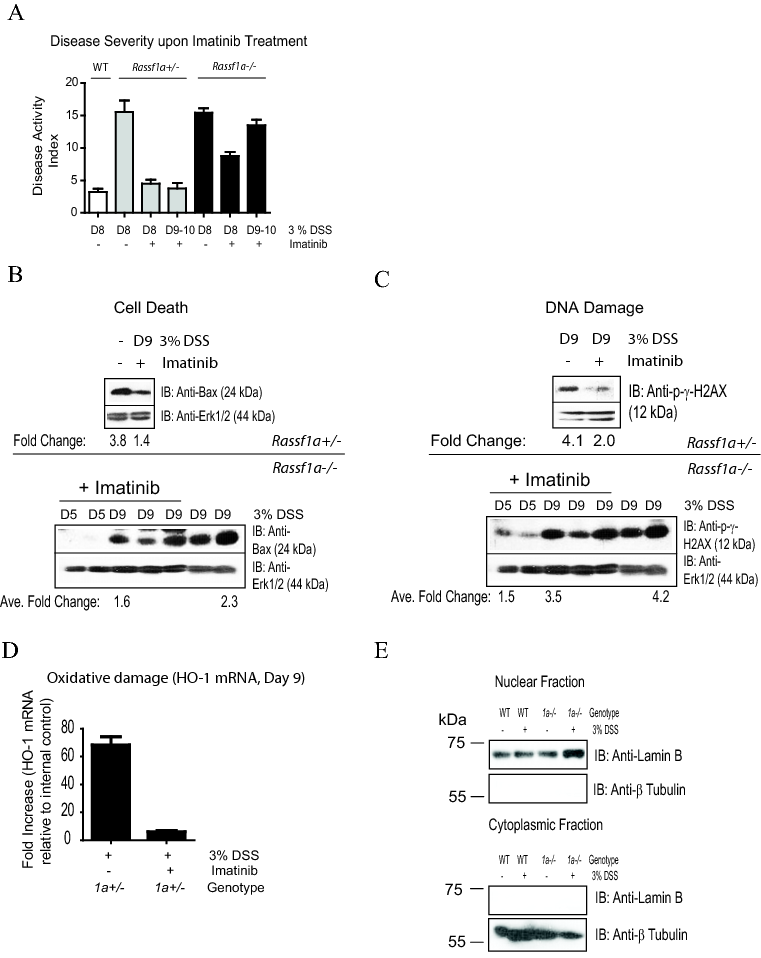

Supplement: S6 Fig — Imatinib was administered intraperitoneally at 60 mg/kg body weight on day 3 and 6 and (A) Disease activity index, (B) cell death using Bax immunoblotting (as an early marker of apoptosis) (in colon lysates), (C) the DNA damage marker phospho-γ-H2AX (in colon lysates) and (D) the oxidative damage marker, HO-1was carried out as indicated (source of sample was colonic mRNA). (E) Purity of our nuclear and cytoplasmic fractions was tested as indicated. (TIF) [file pone.0131150.s001.tif]
