# Supplementary material for: Correction: The Tumor Suppressor Gene, RASSF1A, Is Essential for Protection against Inflammation -Induced Injury
Source: PLoS One. 2015 Jun 24;10(6):e0131150. doi: 10.1371/journal.pone.0131150 (PMC4479583; doi:10.1371/journal.pone.0131150)
Supplement: S7 Fig — (A) PCNA staining with quantitation on the right panel, (B) Detection of pY-YAP was carried out as indicated, (C) pY-YAP immunohistochemistry carried out, (D) Expression of FLAG-YAP (top panel), GST and GST-1A (bottom panel) used in in vitro kinase assay in Fig. 8F.(E) Ubiqutination of p53 was carried out as indicated in colon lysate samples. All baseline (untreated) results not shown were significantly not different from wild type (untreated). (PDF) [file pone.0131150.s002.pdf]

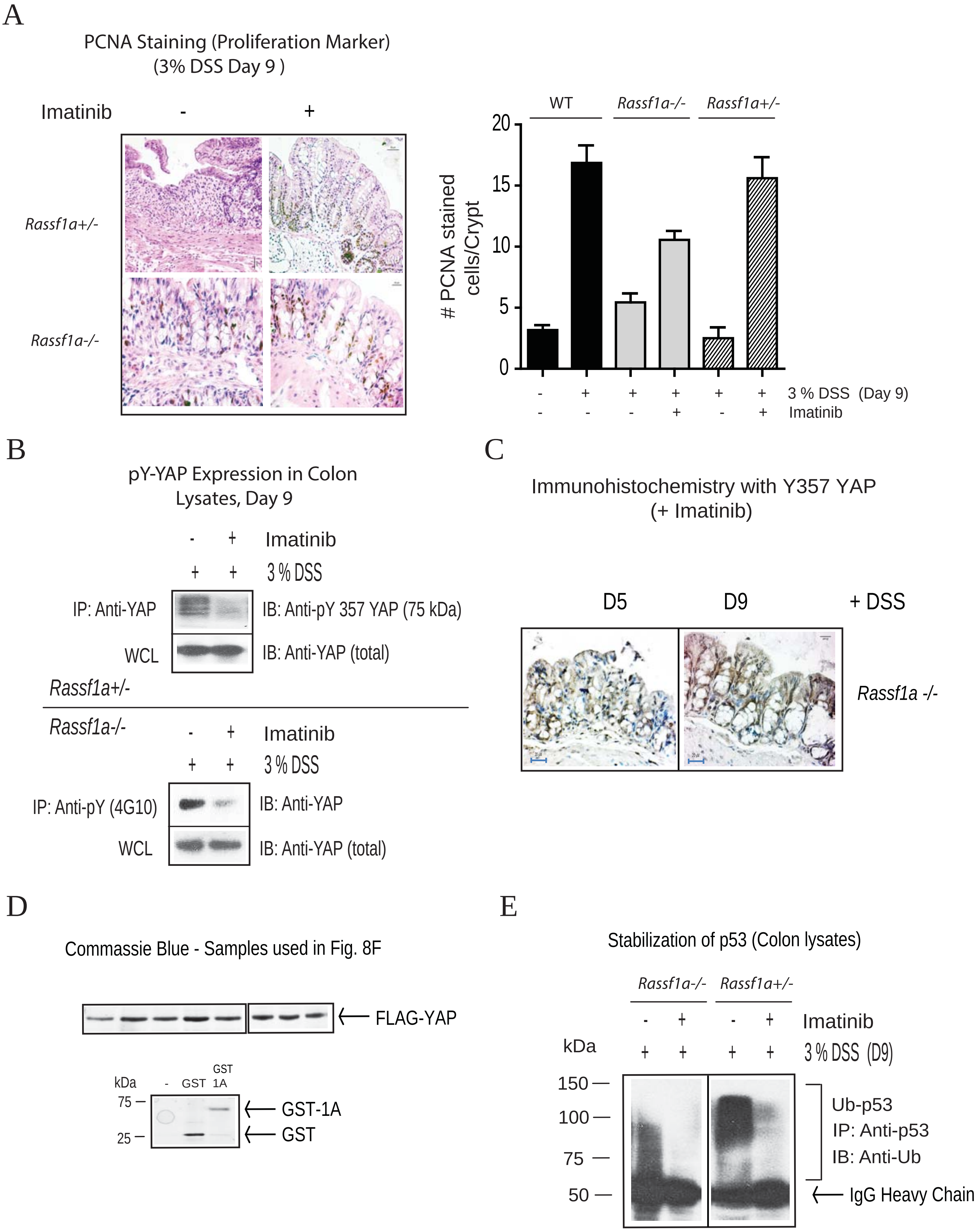
A
PCNA Staining (Proliferation Marker)
(3% DSS Day 9)
Imatinib
-
+
Rassf1a+/-
Rassf1a-/-
WT
Rassf1a-/-
Rassf1a+/-
PCNA stained cells/Crypt
20
15
10
5
0
3 % DSS (Day 9)
Imatinib
B
pY-YAP Expression in Colon Lysates, Day 9
Imatinib
3 % DSS
IP: Anti-YAP
IB: Anti-pY 357 YAP (75 kDa)
WCL
IB: Anti-YAP (total)
Rassf1a+/-
Rassf1a-/-
Imatinib
3 % DSS
IP: Anti-pY (4G10)
IB: Anti-YAP
WCL
IB: Anti-YAP (total)
C
Immunohistochemistry with Y357 YAP
(+ Imatinib)
D5
D9
+ DSS
Rassf1a -/-
D
Commassie Blue - Samples used in Fig. 8F
FLAG-YAP
kDa
-
GST
GST 1A
75
25
GST-1A
GST
E
Stabilization of p53 (Colon lysates)
Rassf1a-/-
Rassf1a+/-
Imatinib
kDa
3 % DSS (D9)
150
100
75
50
Ub-p53
IP: Anti-p53
IB: Anti-Ub
IgG Heavy Chain
